# Correction: Sustained functional benefits after a single set of injections with abobotulinumtoxinA using a 2-mL injection volume in adults with cervical dystonia: 12-week results from a randomized, double-blind, placebo-controlled phase 3b study

**DOI:** 10.1371/journal.pone.0250475

**Published:** 2021-04-14

**Authors:** Atul T. Patel, Mark F. Lew, Khashayar Dashtipour, Stuart Isaacson, Robert A. Hauser, William Ondo, Pascal Maisonobe, Stefan Wietek, Bruce Rubin, Allison Brashear

In Fig 2, the delta and p-value are missing for the last available post-baseline assessment. The missing information reflects a statistically significant difference in the mean TWSTRS total score of aboBoNT-A vs placebo at the last available data point. Please see the correct Fig 2 here.

**Fig 2:**
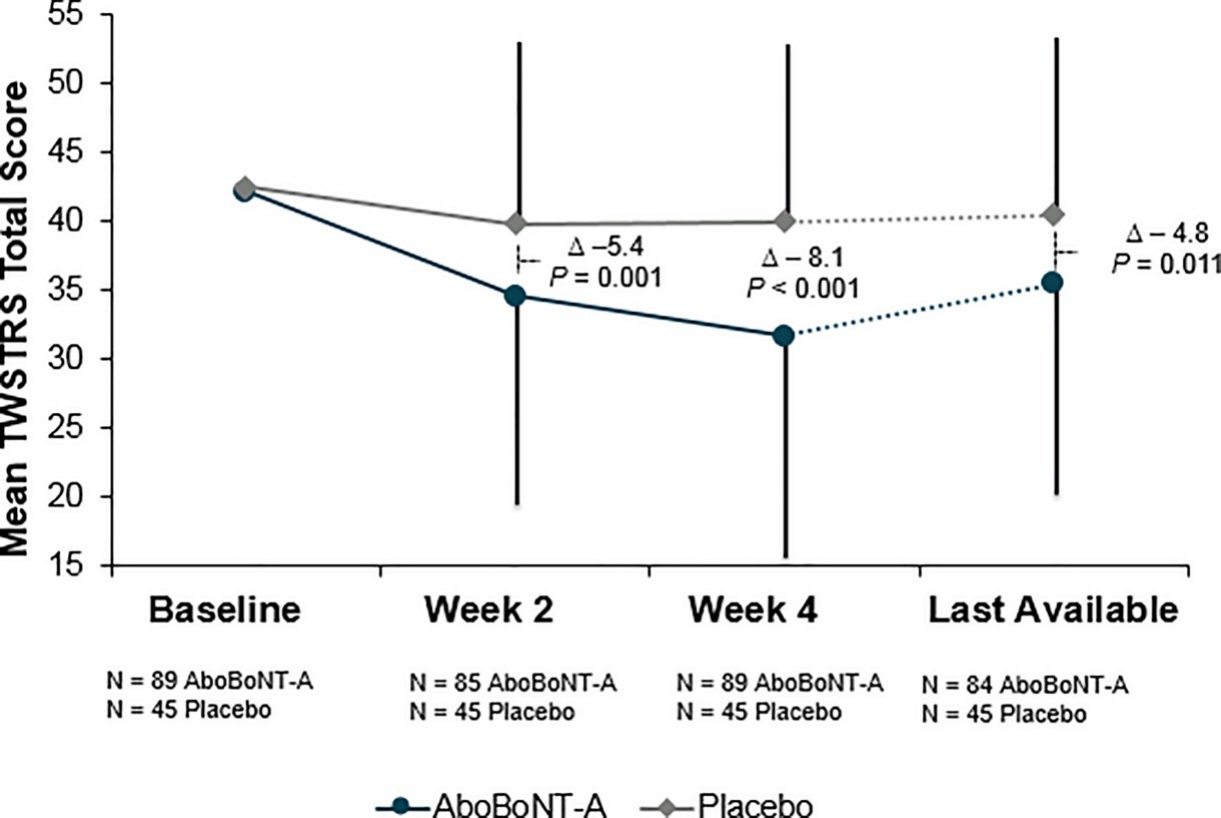
Mean TWSTRS total score. aboBoNT-A = abobotulinumtoxinA; ITT = intent-to-treat (all randomized patients); last available* = last available post-baseline (end of study or early withdrawal); TWSTRS = Toronto Western Spasmodic Torticollis Rating Scale; Δ = weighted overall treatment difference. Error lines indicate standard deviation. *Last available = last available post-baseline (end of study or early withdrawal), mean (SD) study drug exposure: 62.6 (37.8) days.

## References

[1] Patel AT, Lew MF, Dashtipour K, Isaacson S, Hauser RA, Ondo W, et al. (2021) Sustained functional benefits after a single set of injections with abobotulinumtoxinA using a 2-mL injection volume in adults with cervical dystonia: 12-week results from a randomized, double-blind, placebo-controlled phase 3b study. PLoS ONE 16(2): e0245827. 10.1371/journal.pone.0245827 33524060 PMC7850472

