# Supplementary material for: Sustained functional benefits after a single set of injections with abobotulinumtoxinA using a 2-mL injection volume in adults with cervical dystonia: 12-week results from a randomized, double-blind, placebo-controlled phase 3b study
Source: PLoS One. 2021 Feb 1;16(2):e0245827. doi: 10.1371/journal.pone.0245827 (PMC7850472; doi:10.1371/journal.pone.0245827)
Supplement: S1 Fig — (DOCX) [file pone.0245827.s001.docx]

**S1 Fig. Study site locations.**

|  |  |
| --- | --- |
| **United States, Alabama** | |
| University of Alabama at Birmingham |  |
| Birmingham, Alabama, 35294 | |
| **United States, Arizona** | |
| Movement Disorders Center of Arizona, LLC |  |
| Scottsdale, Arizona, 85258 | |
| University of Arizona |  |
| Tucson, Arizona, 85724 | |
| **United States, California** | |
| East Bay Physician’s Group |  |
| Berkeley, California, 94705 | |
| Parkinson’s and Movement Disorder Institute |  |
| Fountain Valley, California, 92708 | |
| Loma Linda University Healthcare, Department of Neurology |  |
| Loma Linda, California, 92354 | |
| USC Keck School of Medicine |  |
| Los Angeles, California, 90033 | |
| UC Davis Medical Center |  |
| Sacramento, California, 95817 | |
| **United States, Colorado** | |
| University of Colorado at Denver Health Sciences |  |
| Aurora, Colorado, 80045 | |
| Advanced Neurosciences Research |  |
| Fort Collins, Colorado, 80528 | |
| **United States, Connecticut** | |
| Associated Neurologists of Southern Connecticut |  |
| Fairfield, Connecticut, 06824 | |
| **United States, District of Columbia** | |
| Georgetown University Hospital |  |
| Washington, District of Columbia, 20007 | |
| **United States, Florida** | |
| Parkinson’s & Movement Disorders Center of Boca Raton |  |
| Boca Raton, Florida, 33486 | |
| University of Florida Center for Movement Disorders and Neurorestoration |  |
| Gainesville, Florida, 32607 | |
| Emerald Coast Center for Neurological Disorders |  |
| Pensacola, Florida, 32514 | |
| Parkinson’s Disease Treatment Center of Southwest Florida |  |
| Port Charlotte, Florida, 33980 | |
| University of South Florida |  |
| Tampa, Florida, 33606 | |
| Guilford Neurologic Associates |  |
| West Palm Beach, Florida, 33407 | |
| Premiere Research Institute at Palm Beach Neurology |  |
| West Palm Beach, Florida, 33407 | |
| **United States, Georgia** | |
| Emory University |  |
| Atlanta, Georgia, 30329 | |
| NeuroTrials Research Inc. |  |
| Atlanta, Georgia,30342 | |
| **United States, Illinois** | |
| Rush University Medical Center |  |
| Chicago, Illinois, 60612 | |
| **United States, Kansas** | |
| Kansas City Bone & Joint Clinic |  |
| Overland Park, Kansas, 66211 | |
| International Clinical Research Institute |  |
| Overland Park, Kansas, 66210 | |
| **United States, Massachusetts** | |
| Tufts Medical Center |  |
| Boston, Massachusetts, 02111 | |
| **United States, Minnesota** | |
| Rehabilitation Consultants PA |  |
| Eagan, Minnesota, 55122 | |
| **United States, New Jersey** | |
| University of Medicine and Dentistry of New Jersey |  |
| Stratford, New Jersey, 08084 | |
| Atlantic Neuroscience Institute |  |
| Summit, New Jersey, 07901 | |
| **United States, New York** | |
| Kingston Neurological Associates |  |
| Kingston, New York, 12401 | |
| Fazzini Parkinson’s Disease & Dystonia Center |  |
| New York, New York, 10016 | |
| The Ichan School of Medicine at Mount Sinai |  |
| New York, New York, 10029 | |
| Island Neurological Associates |  |
| Plainview, New York, 11803 | |
| **United States, North Carolina** | |
| Guilford Neurologic Associates; Cone Health Medical Group |  |
| Greensboro, North Carolina, 27405 | |
| Wake Forest School of Medicine |  |
| Winston-Salem, North Carolina, 27157 | |
| **United States, Ohio** | |
| University of Cincinnati Physicians Company, LLC |  |
| Cincinnati, Ohio, 45267 | |
| **United States, Oregon** | |
| OHSU Center for Health and Healing |  |
| Portland, Oregon, 97239 | |
| **United States, Pennsylvania** | |
| Penn State Hershey Neurology |  |
| Hershey, Pennsylvania, 17033 | |
| **United States, South Carolina** | |
| Coastal Neurology |  |
| Port Royal, South Carolina, 29935 | |
| **United States, Texas** | |
| North Texas Movement Disorders Institute |  |
| Bedford, Texas, 76201 | |
| Baylor College of Medicine |  |
| Houston, Texas, 77030 | |
| University of Texas Health Science Center at Houston |  |
| Houston, Texas, 77030 | |
| **United States, Washington** | |
| Puget Sound Neurology |  |
| Tacoma, Washington, 98409 | |
