# Supplementary figures and images for: Sustained functional benefits after a single set of injections with abobotulinumtoxinA using a 2-mL injection volume in adults with cervical dystonia: 12-week results from a randomized, double-blind, placebo-controlled phase 3b study
Source: PLoS One. 2021 Feb 1;16(2):e0245827. doi: 10.1371/journal.pone.0245827 (PMC7850472; doi:10.1371/journal.pone.0245827)

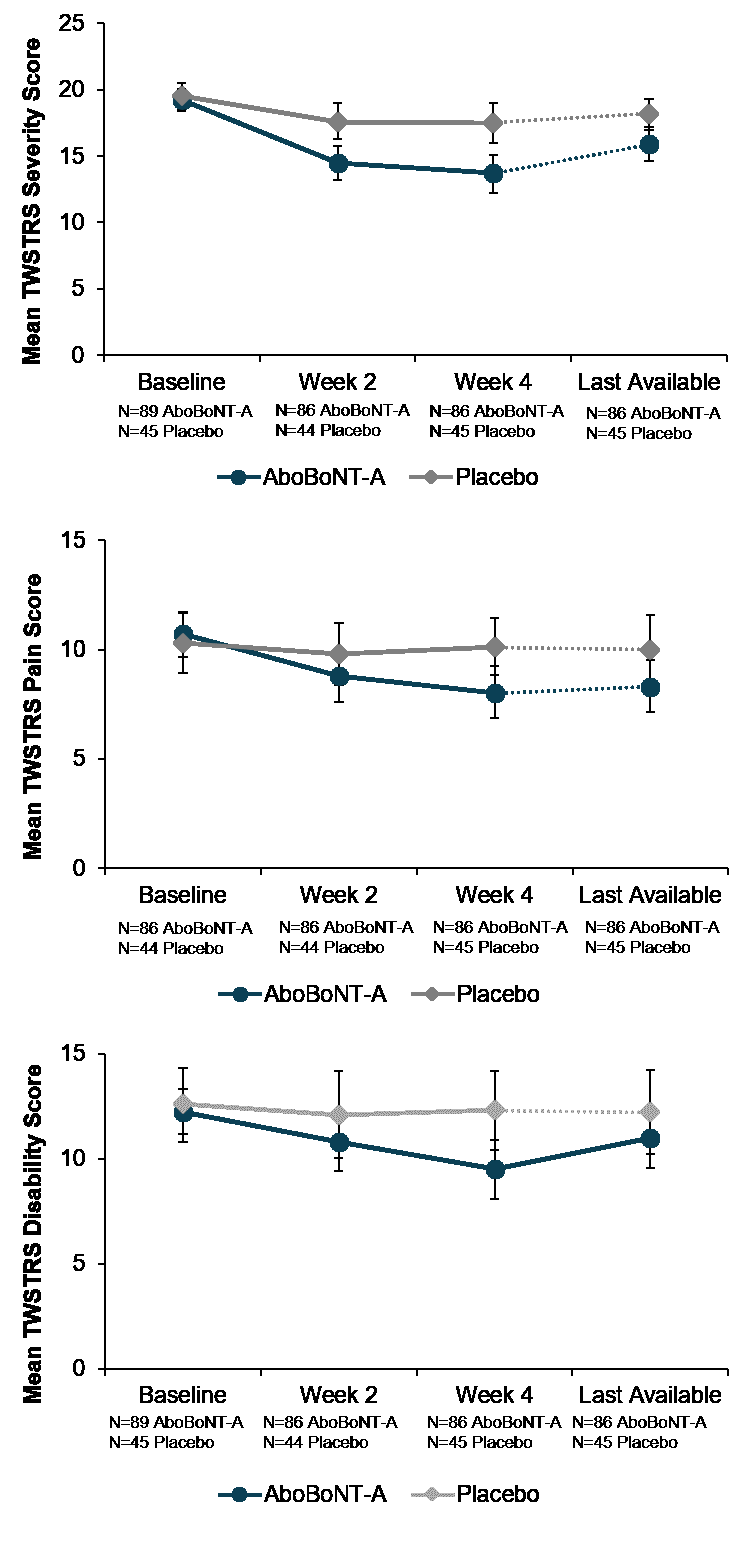

Supplement: S2 Fig — Mean TWSTRS sub-scale scores across time points (ITT population): A) Mean TWSTRS severity score across time points. B) Mean TWSTRS pain score across time points. C) Mean TWSTRS disability score across time points. Tertiary endpoints: no formal statistical testing was conducted. ITT = intent-to-treat (all randomized patients); last available = last available post-baseline* (end of study or early withdrawal); TWSTRS = Toronto Western Spasmodic Torticollis Rating Scale. Error bars represent the 95% confidence interval. *Last available = last available post-baseline (end of study or early withdrawal), mean (SD) study drug exposure: 62.6 (37.8) days. (PNG) [file pone.0245827.s002.png]

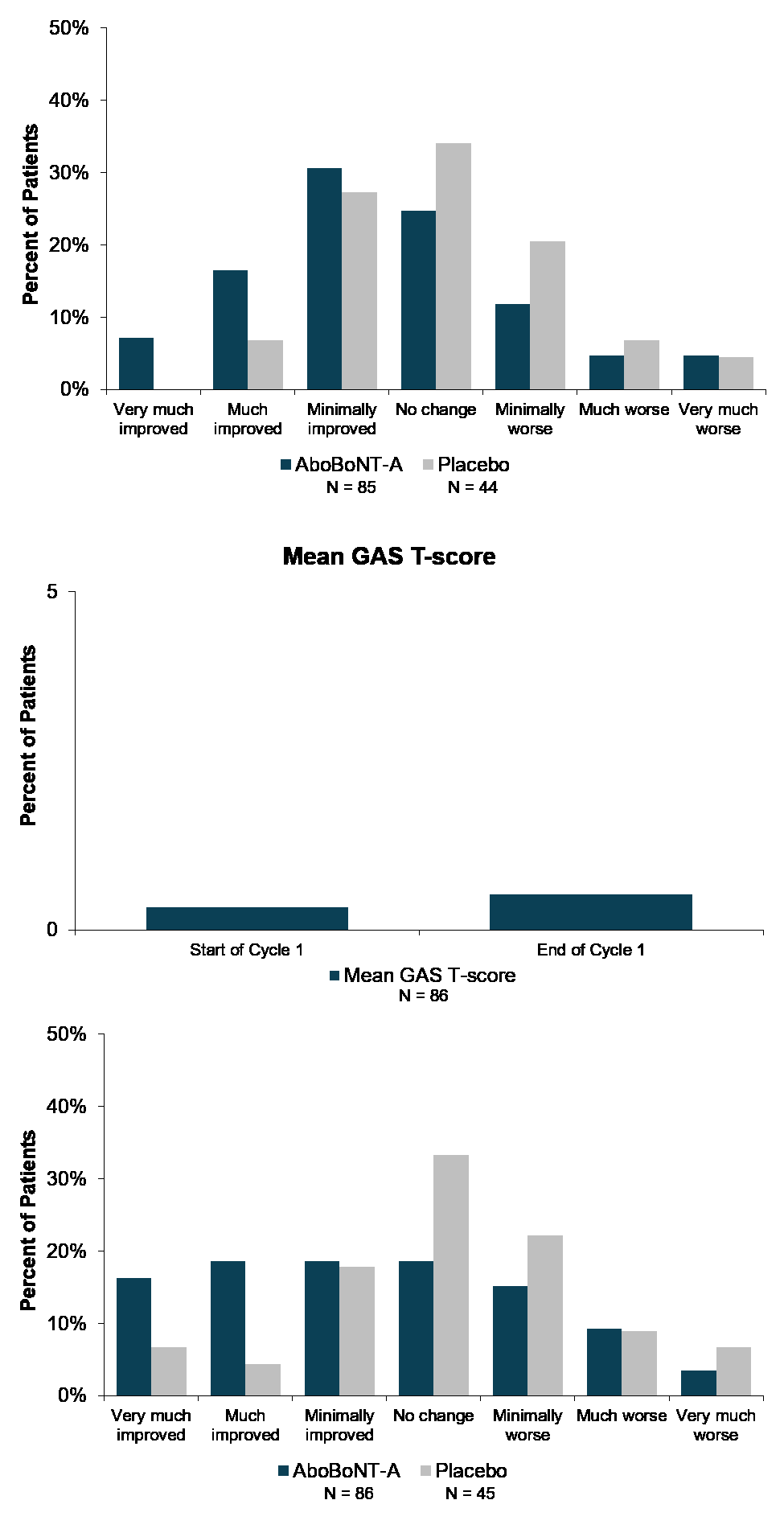

Supplement: S3 Fig — Patient Global Impression of Change at (A) Week 2, (B) Week 4, and (C) last available (ITT). A. Week 2. B. Week 4. C. Last available. *Last available = last available post-baseline (end of study or early withdrawal), mean (SD) study drug exposure: 62.6 (37.8) days. (PNG) [file pone.0245827.s003.png]
