# Supplementary material for: Sustained functional benefits after a single set of injections with abobotulinumtoxinA using a 2-mL injection volume in adults with cervical dystonia: 12-week results from a randomized, double-blind, placebo-controlled phase 3b study
Source: PLoS One. 2021 Feb 1;16(2):e0245827. doi: 10.1371/journal.pone.0245827 (PMC7850472; doi:10.1371/journal.pone.0245827)
Supplement: S1 File — (PDF) [file pone.0245827.s004.pdf]

**PROTOCOL: FINAL: Version 6.0, 27 March 2014**

# A PHASE IIIb, MULTICENTRE, RANDOMISED, DOUBLE-BLIND, PLACEBO-CONTROLLED STUDY EVALUATING THE EFFICACY AND SAFETY OF DYSPORT USING 2 mL DILUTION IN ADULTS WITH CERVICAL DYSTONIA

**STUDY PROTOCOL**
**Study number: A-TL-52120-169**
**Dysport**
**Final Version 6.0: 27 March 2014**
**(Incorporating Amendment 5)**

**Sponsor's Medically Responsible Person:**
Kathleen Graham Lomax, MD
Senior Medical Director
106 Allen Road,
Basking Ridge, NJ 07920, USA
Tel: + 1 908 275 6402
Fax: + 1 908 275 6301

**Study Sponsor:**
Ipsen Biopharmaceuticals, Inc
106 Allen Road,
Basking Ridge, NJ 07920, USA
Tel: + 1 908 275 6300
Fax: + 1 908 275 6301

**Monitoring Office**:
Angela Castagna
Senior Clinical Project Manager,
Ipsen Biopharmaceuticals, Inc
Medical Affairs
106 Allen Road,
Basking Ridge,
NJ 07920, USA
Tel: + 1 908 275 6421
Fax: + 1 908 275 6301

**Co-ordinating Investigator:**
Mark F. Lew, MD, FAAN
Director, Division of Movement Disorders
Vice-Chair, Department of Neurology
Keck/USC School of Medicine, USCHCC2
1520 San Pablo St #3000, Los Angeles,
CA 90033, USA
Tel: + 1 323 422 5728
Fax: + 1 323 422 5794

**Pharmacovigilance/Emergency Contact (USA only):**
Kathleen Graham Lomax, MD, Senior Medical Director
106 Allen Road, Basking Ridge, NJ 07920, USA
Tel: + 1 908 275 6402- during office hours
+ 1 973 943 6229 - mobile telephone for emergencies
Fax for SAE reporting: + 1 888 525 2419

*Persons supplied with this information must understand that it is **strictly confidential**. Information contained herein cannot be disclosed, submitted for publication or used for any purpose other than that contemplated herein without the Sponsor's prior written authorisation.*

# PROTOCOL SIGNATURES

**Investigator Signature:**

I have read and agree to the protocol A-TL-52120-169, a phase IIIb, multicentre, randomised, double-blind, placebo-controlled study evaluating the efficacy and safety of Dysport using 2 mL dilution in adults with cervical dystonia. I am aware of my responsibilities as an Investigator under the guidelines of Good Clinical Practice (GCP)[1], local regulations (as applicable) and the study protocol. I agree to conduct the study according to these guidelines and to appropriately direct and assist the staff under my control, who will be involved in the study.

NAME: ____________________

TITLE: [PRINCIPAL] INVESTIGATOR: SIGNATURE: ____________________

DATE: ____________________

OFFICE:

Full investigational site contact details, including telephone numbers, will be documented in the Trial Master File.

**On behalf of the Sponsor:**

NAME: **Kathleen Graham Lomax, MD** ____________________

TITLE: Senior Medical Director SIGNATURE: ____________________

DATE: ____________________

OFFICE:

Note: For IND studies, a Form 1572 will also be completed.

[1] ICH Harmonised Tripartite Guideline E6: Note for Guidance on Good Clinical Practice (CPMP/ICH/135/95) Step 5, adopted by CPMP July 1996.
Food and Drug Administration Code of Federal Regulations for Good Clinical Practices Parts 50, 56, 312, 314

# SYNOPSIS

| | |
|---|---|
| **Study Title:** | A phase IIIb, multicentre, randomised, double-blind, placebo-controlled study evaluating the efficacy and safety of Dysport using 2 mL dilution in adults with cervical dystonia |
| **Study Objectives:** | **Primary Study Objective**<br>The primary objective of the study is to evaluate the efficacy of Dysport 500 units (U)/vial using 2 mL dilution compared with placebo for the treatment of cervical dystonia (CD).<br>**Secondary Study Objectives**<br>The secondary study objectives are to:<br>(1) Evaluate the safety of Dysport 500 U/vial using 2 mL dilution compared with placebo for the treatment of CD.<br>(2) Evaluate Investigator's global assessment of improvement.<br>(3) Evaluate subject-related outcomes:<br>• Onset of symptom improvement.<br>• The effect on pain.<br>• The effect on changes from baseline in health-related quality of life.<br>• Satisfaction with treatment. |
| **Phase of Study:** | Phase IIIb |
| **Study Design:** | This is a phase IIIb, multicentre, randomised, double-blind, placebo-controlled study evaluating the efficacy and safety of Dysport 500 U/vial using a 2 mL dilution in adults with CD. In this study, the treatment dose of Dysport is between 250 U and 500 U. The study will be conducted at approximately 45 centres in the United States of America.<br>All subjects are planned to have a single treatment in this study. The overall study duration will be approximately 13 months and will include subject recruitment (approximately 9 months) and last subject follow up (up to 4 months). Individual subject treatment duration will be between 4 and 16 weeks.<br>At study entry, subjects will be randomised in a ratio of 2:1 to receive either Dysport or placebo. The randomisation will be stratified according to whether the subject is botulinum toxin (BTX) treatment naïve or has been successfully treated with Botox based on Investigator judgment (non-naïve to BTX treatment).<br>If any oral medication given for CD is initiated prior to study entry, the regimen should be continued at the same frequency and dose until at least the Week 4 visit and, whenever possible, until the end of the study.<br>Subjects will have screening, eligibility, and baseline assessments conducted prior to study treatment. The screening visit and baseline visit may be on the same day or within 7 days of each other. Following study treatment, follow up visits will be performed at Week 2, Week 4 and Week 12 (+28 days/4 weeks) or early withdrawal due to any reason. |

| | |
|---|---|
| | All subjects who complete the Week 12 visit will be considered to have completed the study and will be offered entry into an open-label extension (OLE) study, which consists of up to three treatment cycles of Dysport using the 2 mL dilution scheme. Between Weeks 4 and 8, subjects may be deemed eligible for early entry into the OLE study, i.e. before they reach the planned Week 12 study visit. In order to be eligible for early entry into the OLE study, subjects must fulfil the following criteria:<br>• At the scheduled Week 4 visit, or at an unscheduled visit occurring between Weeks 4 to 8, the Toronto Western Spasmodic Torticollis Rating Scale (TWSTRS) total score as compared to baseline is reduced by ≤15%.<br>• Have no ongoing adverse events (AEs), which in the opinion of the Investigator are related to study treatment and that preclude the Subject from receiving continuing therapy.<br>• All other OLE study entry criteria. |
| **Study Population:** | Approximately 132 male and female subjects with CD will be enrolled.<br>**Inclusion Criteria:**<br>All subjects must fulfil the following:<br>(1) Written informed consent prior to any study related procedures.<br>(2) Male or female subjects ≥18 years of age.<br>(3) Primary diagnosis of idiopathic CD at least 9 months since onset, and either:<br>• Previously untreated with BTX (naïve to BTX), or<br>• Currently treated with Botox at a total dosing range of 100 to 200 U and ≤60 U in the sternocleidomastoid (SCM) muscle at the last injection cycle, and having had a satisfactory treatment response in the enrolling Investigator's judgment during the last two sequential cycles of Botox within the past 18 months for the treatment of CD.<br>(4) For non-naïve BTX subjects, a minimum interval of 12 weeks since the last Botox injection and having returned at least to their typical pre-treatment status as assessed by the Investigator.<br>(5) Non-naïve subjects may have received any other formulations of BTX prior to study entry as long as they have had a satisfactory clinical response to the last two injection sessions that must have been Botox.<br>(6) TWSTRS meeting the following criteria at baseline:<br>• TWSTRS-total score ≥20.<br>• TWSTRS-severity subscale score >10.<br>**Exclusion Criteria:**<br>Subjects will be excluded if any of the following apply:<br>(1) In apparent remission from CD.<br>(2) Diagnosis of pure retrocollis or pure anterocollis. |

| | |
|---|---|
| | (3) For non-naïve subjects, previous poor response (as determined by standard practice) to either of the last two Botox treatments for CD.<br>(4) Known requirement of <100 U or >200 U Botox injected into the neck muscles.<br>(5) Known resistance to any botulinum toxin type A (BTX-A).<br>(6) Lack of therapeutic response to previous Dysport treatments for CD.<br>(7) Requirement for BTX injection to site(s) for disorders other than CD and unable to avoid such treatment(s) for the duration of the study.<br>(8) Known hypersensitivity to BTX or related compounds, or any component in the study drug formulation.<br>(9) Allergy to cow's milk protein.<br>(10) Myasthenia gravis, other disease of the neuromuscular junction or clinically significant, persistent neuromuscular weakness, or disease or symptoms that can interfere with the TWSTRS scoring.<br>(11) Total body weight <95 lbs (43.09 kg).<br>(12) Previous phenol injections to the neck muscles.<br>(13) Previous myotomy or denervation surgery involving the neck or shoulder region or deep brain stimulation to treat CD.<br>(14) Cervical contracture that limits passive range of motion.<br>(15) Physiotherapy initiated less than 4 weeks before study entry or expected to be initiated during the study.<br>(16) Treatment with aminoglycoside antibiotics within the last 30 days prior to study treatment.<br>(17) Current or expected requirement for concomitant medication that may interfere with the evaluation of study treatment (note: muscle relaxants, narcotics, and benzodiazepines are permitted if the dosage has been stable for the 4 weeks prior to study treatment and is expected to remain at this dose until the Week 4 assessment. Every effort should be made to keep concomitant CD treatment constant throughout the study. However, changes in pain medication are acceptable if absolutely necessary according to clinical judgment).<br>(18) Received any investigational new drug or device within 30 days prior to inclusion in the study.<br>(19) Pregnant and/or lactating females.<br>(20) Females of childbearing potential with a positive pre-study urine pregnancy test (a positive urine pregnancy test can be confirmed by serum pregnancy test at the discretion of the Investigator) and subjects, or their partners, who do not agree to use adequate contraception (hormonal or barrier method of birth control) prior to injection of study treatment and for the duration of study participation. Non-childbearing potential is |

| | |
|---|---|
| | defined as post-menopause for at least 1 year, surgical sterilisation at least 3 months before entering screening, or hysterectomy.<br>(21) Individuals with family or employee relationship to investigative site staff or Sponsor staff involved in the conduct of the study.<br>(22) Any medical condition that compromises compliance with the objectives and procedures of this protocol or precludes the administration of BTX, including swallowing and other respiratory abnormality, as judged by the Investigator.<br>(23) In the opinion of the Investigator the subject is unable and/or unwilling to comply fully with the protocol and the study instructions. |
| **Study Treatment:** | **Study Drug:**<br>Dysport will be supplied in a clear glass vial as a freeze-dried white powder containing nominally 500 U of BTX-A-haemagglutinin complex together with 125 µg of human serum albumin and 2.5 mg of lactose.<br>**Control Compound:**<br>Placebo will be supplied in identical clear glass vials, containing 125 µg of human serum albumin and 2.5 mg of lactose.<br>All of the study treatment will be similar in size, colour, smell, taste and appearance allowing the blinded conditions of the study to be maintained. Before administration, the Dysport and placebo vials will be reconstituted at the investigational site with 2 mL preservative free sodium chloride for injection (0.9%). Detailed instructions will be provided for the volume which needs to be withdrawn from the reconstituted Dysport vials. At study entry, subjects will be randomised 2:1 into one of two study treatment groups (Dysport or placebo). All eligible subjects will receive either Dysport or placebo by intramuscular injection. The dose of Dysport will be between 250 and 500 U.<br>Subjects randomised to the Dysport group and who are naïve to BTX treatments for CD will receive 500 U of Dysport in a minimum of two clinically affected neck muscles. The sites of injection and the dose per site will be determined by the Investigator according to the standard practice and disease presentation. Non-naïve subjects who are randomised to the Dysport group will receive a dose up to 500 U of Dysport and based on the ratio of 2.5:1 (Dysport:Botox from previous treatment dose for CD). Subjects in this group will receive Dysport injected into the same neck muscles that were injected during the last two sequential cycles of Botox within the past 18 months for the treatment of CD. The amount of study treatment injected into the SCM muscle should be limited to ≤0.6 mL (150 U Dysport).<br>Electromyography may be used to identify the sites of injection for |

| | |
|---|---|
| | any subjects. |
| **Study Evaluations:** | **Efficacy Evaluations**:<br>• The TWSTRS total score as assessed by the Investigator at baseline and at each post-treatment visit (including the early withdrawal visit) to the study centre.<br>• Investigator's assessment of TWSTRS severity, disability and pain subscale scores at baseline and at each post-treatment visit (including the early withdrawal visit) to the study centre.<br>• Subject's assessment of pain at baseline and at each post-treatment visit (including the early withdrawal visit) to the study centre. The pain assessment will be made using a Numeric Rating Scale (NRS), describing the pain in the last 24 hours prior to assessment. The NRS has a range from 0 (no pain) to 10 (worst possible pain).<br>• Subject's assessment of pain at baseline and at each post-treatment visit (including the early withdrawal visit) to the study centre using the Brief Pain Inventory (BPI) short form.<br>• Subject's assessment of depression at baseline and at each post-treatment visit (including the early withdrawal visit) to the study centre using the Patient Health Questionnaire (PHQ)-9.<br>• Investigator's and subject's global assessment of change of CD at each post-treatment visit (including the early withdrawal visit) to the study centre.<br>• Quality of life assessed at baseline and at each post-treatment visit (including the early withdrawal visit) to the study centre using the Cervical Dystonia Impact Profile (CDIP)-58 questionnaire.<br>• Subject's overall satisfaction with treatment will be assessed at each post-treatment visit (including the early withdrawal visit) to the study centre.<br>**Safety Evaluations:**<br>• Collection of AEs at each study visit.<br>• Assessment of suicidality (suicidal ideation and behaviour) using the Columbia-Suicide Rating Severity Scale (C-SSRS) at each study visit.<br>• Monitoring of vital signs (blood pressure, heart rate, oral temperature and respiratory rate) at screening and at the end of study or early withdrawal visit.<br>• Clinical laboratory evaluations (biochemistry only) at screening and at the end of study or early withdrawal visit.<br>• Physical examination at screening and at the end of study or early withdrawal visit. |
| **Study Endpoints:** | **Primary Efficacy Endpoint:**<br>• Change from baseline in TWSTRS total score at the Week 4 visit.<br>**Secondary Efficacy Endpoints:** |

| | |
|---|---|
| | • Change from baseline in TWSTRS total score at the Week 2 visit.<br>• Clinical Global Impression of Change (CGIC) assessment of CD at the Week 2 and Week 4 visits.<br>• Treatment response at the Week 2 and Week 4 visits. A treatment responder is defined as a subject who had at least a 30% reduction in the TWSTRS total score after treatment.<br>• Change from baseline in CDIP-58 score at the Week 2 and Week 4 visits.<br>**Tertiary Efficacy Endpoints:**<br>• Change from baseline in TWSTRS severity, disability and pain subscale scores at the Week 2, 4 and 12 visits.<br>• Change from baseline in TWSTRS total score at the Week 12 visit.<br>• Patient Global Impression of Change (PGIC) assessment of CD change at the Week 2, 4 and 12 visits.<br>• Change from baseline in pain NRS at the Week 2, 4 and 12 visits.<br>• Change from baseline in BPI short form at the Week 2, 4 and 12 visits.<br>• Change from baseline in PHQ-9 at the Week 2, 4 and 12 visits.<br>• Modified Treatment Satisfaction Questionnaire for Medication (TSQM)-9 at the Week 2, 4 and 12 visits.<br>• Clinical Global Impression of Change at the Week 12 visit.<br>• Treatment response at the Week 12 visit.<br>• Change from baseline in CDIP-58 at the Week 12 visit.<br>**Safety Endpoints:**<br>• Treatment emergent AEs (TEAEs) (including information on seriousness, intensity, drug relationship and TEAEs leading to study withdrawal) from the granting of informed consent and at each study visit.<br>• Suicidal ideation and behaviour will be assessed using the C-SSRS at each study visit.<br>• Absolute values and changes from baseline in vital signs, clinical laboratory parameters (biochemistry only) and physical examination findings at screening and at the end of study or early withdrawal visits. |
| **Statistical Methods:** | The intent to treat (ITT) population will include all randomised subjects. The modified intent to treat (mITT) population is a subset of the ITT population, composed of randomised subjects with both a baseline and a Week 4 post-treatment TWSTRS total score assessment.<br>The per protocol population will include all subjects in the mITT population who were not major protocol violators.<br>The population for the safety analyses will be composed of all randomised subjects who received study treatment regardless of the |

| | |
|---|---|
| | amount of study treatment administered and had at least one follow up safety assessment..<br>The primary efficacy endpoint is the change from baseline in TWSTRS total score at the Week 4 visit. The efficacy of Dysport 2 mL will be demonstrated by the testing of superiority of Dysport 2 mL to placebo at a two-tailed significance level of 0.05.<br>**Sample Size Calculation**<br>The number of 132 randomised subjects (i.e. 88 subjects in the Dysport group and 44 subjects in the placebo group) is considered sufficient to demonstrate the superiority of Dysport to placebo assuming a minimum clinically relevant difference in the adjusted least squares mean change from baseline in TWSTRS total score at Week 4 between Dysport 2 mL and placebo equal to 5.5, a common standard deviation in the change from baseline in TWSTRS total score at Week 4 equal to 8.8, a power of 90%, a two-tailed type I error equal to 0.05 and 10% drop out rate. |

# TABLE OF CONTENTS

1 LIST OF ABBREVIATIONS AND DEFINITION OF TERMS ..... 14
2 INTRODUCTION ..... 16
2.1 Disease Review ..... 16
2.2 Compound Review ..... 17
2.3 Clinical Trial Rationale ..... 17
3 STUDY OBJECTIVES ..... 18
3.1 Primary Study Objective ..... 18
3.2 Secondary Study Objectives ..... 18
4 STUDY DESIGN ..... 19
4.1 Overview ..... 19
*4.1.1 Population Characteristics ..... 20*
*4.1.2 Design ..... 20*
*4.1.3 Structure ..... 20*
*4.1.4 Stopping Rules and Discontinuation Criteria ..... 21*
*4.1.5 Early Study Termination ..... 21*
4.2 Endpoints ..... 21
*4.2.1 Efficacy Endpoints ..... 21*
*4.2.1.1 Primary Efficacy Endpoint ..... 21*
*4.2.1.2 Secondary Efficacy Endpoints ..... 21*
*4.2.1.3 Tertiary Efficacy Endpoints ..... 21*
*4.2.2 Safety Endpoints ..... 22*
4.3 Justification of Design ..... 22
*4.3.1 Study Population for Analysis ..... 22*
*4.3.2 Study Duration ..... 22*
5 COMPLIANCE WITH GOOD CLINICAL PRACTICE, ETHICAL CONSIDERATIONS & INFORMED CONSENT ..... 23
5.1 Compliance with Good Clinical Practice and Ethical Considerations ..... 23
5.2 Informed Consent ..... 23
6 STUDY POPULATION ..... 24
6.1 Screening Log and Number of Subjects ..... 24
6.2 Inclusion Criteria ..... 24
6.3 Exclusion Criteria ..... 25
6.4 Subject Withdrawal Criteria ..... 26
6.5 Discontinuation/Withdrawal Procedures ..... 26
7 METHODOLOGY ..... 26
7.1 Study Schedule ..... 26
7.2 Study Visits ..... 28
*7.2.1 Pre-study Screening (Day -7 to Day -1, Visit 1) ..... 28*
*7.2.2 Baseline (Day 1, Visit 2) ..... 28*
*7.2.3 Post-treatment Follow Up (Week 2+2 days, Visit 3 and Week 4±2 days, Visit 4) ..... 28*
*7.2.4 Study Completion or Early Withdrawal (Week 12+28 days, Visit 5) ..... 29*
8 STUDY EVALUATIONS ..... 29
8.1 Efficacy Endpoints and Evaluations ..... 29

***8.1.1 Primary Efficacy Endpoint and Evaluation......29***
***8.1.2 Secondary Efficacy Endpoints and Evaluations......30***
*8.1.2.1 Toronto Western Spasmodic Torticollis Rating Scale......30*
*8.1.2.2 Clinical Global Impression of Change......30*
*8.1.2.3 Cervical Dystonia Impact Profile-58......30*
***8.1.3 Tertiary Efficacy Endpoints and Evaluations......30***
*8.1.3.1 Toronto Western Spasmodic Torticollis Rating Scale......30*
*8.1.3.2 Patient Global Impression of Change......30*
*8.1.3.3 Numeric Rating Scale: Subject Assessment of Pain......31*
*8.1.3.4 Brief Pain Inventory......31*
*8.1.3.5 Patient Health Questionnaire......31*
*8.1.3.6 Modified Treatment Satisfaction Questionnaire for Medication......31*
*8.1.3.7 Clinical Global Impression of Change......31*
*8.1.3.8 Cervical Dystonia Impact Profile-58......31*
**8.2 Safety Endpoints and Evaluations......31**
***8.2.1 Adverse Events......32***
***8.2.2 Physical Examination......32***
***8.2.3 Vital Signs......32***
***8.2.4 Electrocardiogram......32***
***8.2.5 Clinical Laboratory Tests......32***
***8.2.6 Suicidality......33***
**8.3 Total Blood Volume......33**
**8.4 Pharmacodynamic Endpoints and Evaluations......33**
**8.5 Pharmacokinetic Endpoints and Evaluations......33**
**8.6 Blood Samples for Putative Antibodies......33**
**9 STUDY TREATMENTS......33**
**9.1 Study Treatments Administered......33**
***9.1.1 Dysport......33***
***9.1.2 Placebo......34***
***9.1.3 Administration Procedures......34***
**9.2 Subject Identification and Allocation to Study Treatment......35**
***9.2.1 Randomisation......35***
***9.2.2 Blinding, Emergency Envelopes and Breaking the Blind......36***
**9.3 Study Treatment Supply, Packaging and Labelling......36**
**9.4 Compliance......37**
**9.5 Study Treatment Storage and Accountability......37**
**9.6 Concomitant Medication/Therapy......37**
**9.7 Treatment of Overdose of IMP......37**
**10 ADVERSE EVENT REPORTING......38**
**10.1 Disease Progression......38**
**10.2 Categorisation of Adverse Events......38**
***10.2.1 Intensity Classification......38***
***10.2.2 Causality Classification......38***
***10.2.3 Assessment of Expectedness......39***
***10.2.4 Laboratory Test Abnormalities......39***
***10.2.5 Abnormal Physical Examination Findings......39***

*10.2.6 Suicidality Assessment ... 39*
*10.2.7 Other Investigation Abnormal Findings ... 40*
**10.3 Recording and Follow up of Adverse Events ... 40**
**10.4 Serious Adverse Events ... 40**
*10.4.1 Definitions ... 40*
*10.4.2 Reporting Requirements ... 41*
*10.4.3 Mandatory Information for Reporting an SAE ... 41*
*10.4.4 Reporting Exemptions ... 42*
**10.5 Pregnancy ... 42**
**10.6 Deaths ... 42**
**10.7 Discontinuation/Withdrawal due to Adverse Events/Serious Adverse Events ... 43**
**10.8 Reporting to IRBs/Other Investigators ... 43**
11 STATISTICAL CONSIDERATIONS ... 43
**11.1 Subject Classification and Definitions ... 43**
**11.2 Analyses Populations Definitions ... 44**
*11.2.1 Populations Analysed ... 44*
*11.2.2 Subject Allocation and Reasons for Exclusion from the Analyses ... 44*
**11.3 Sample Size Determination ... 44**
*11.3.1 Significance Testing and Estimations ... 45*
**11.4 Statistical/Analytical Methods ... 45**
*11.4.1 Demographic and Other Baseline Characteristics ... 45*
*11.4.1.1 Homogeneity of Treatment Groups ... 45*
*11.4.1.2 Subject Disposition and Withdrawals ... 45*
*11.4.2 Pharmacokinetic Data ... 45*
*11.4.3 Efficacy Evaluation ... 45*
*11.4.4 Adjustment for Country/Centre Effect ... 47*
*11.4.5 Safety Evaluation ... 47*
**11.5 Subgroup Analyses ... 48**
**11.6 Interim Analyses and Data Monitoring ... 49**
**11.7 Final Analysis ... 49**
12 MONITORING PROCEDURES ... 49
**12.1 Routine Monitoring ... 49**
13 STUDY MANAGEMENT ... 49
**13.1 Inspections and Auditing Procedures ... 49**
**13.2 Data Recording of Study Data ... 49**
**13.3 Source Data Verification ... 50**
**13.4 Data Quality ... 51**
**13.5 Data Management ... 51**
**13.6 Study Management Committees ... 52**
*13.6.1 Steering Committee ... 52*
**13.7 Record Archiving and Retention ... 52**
14 ADMINISTRATION PROCEDURES ... 52
**14.1 Regulatory Approval ... 52**
**14.2 Publication Policy ... 53**
**14.3 Clinical Study Report ... 53**
**14.4 Contractual and Financial Details ... 53**

14.5 Insurance, Indemnity and Compensation ..... 54
15 PROTOCOL AMENDMENTS ..... 54
16 REFERENCES ..... 55
17 LIST OF APPENDICES ..... 57

# LIST OF TABLES

Table 1 Schedule of Assessments ..... 27

# LIST OF FIGURES

Figure 1 Study Flow Chart ..... 19

# 1 LIST OF ABBREVIATIONS AND DEFINITION OF TERMS

| ABBREVIATION | Wording Definition |
|---|---|
| **AE** | Adverse Event |
| **ANOVA** | Analysis of Variance |
| **ANCOVA** | Analysis of Covariance |
| **BP** | Blood Pressure |
| **BPI** | Brief Pain Inventory |
| **BTX** | Botulinum Toxin |
| **BTX-A** | Botulinum Toxin Type A |
| **BTX-A-HAC** | Botulinum Type A Toxin Haemagglutinin Complex |
| **CD** | Cervical Dystonia |
| **CDIP-58** | Cervical Dystonia Impact Profile-58 |
| **CGIC** | Clinical Global Impression of Change |
| **CRO** | Contract Research Organisation |
| **CSR** | Clinical Study Report |
| **C-SSRS** | Columbia-Suicide Severity Rating Scale |
| **CTSU** | Clinical Trial Supplies Unit (relates to Sponsor) |
| **Da** | Dalton |
| **eCRF** | Electronic Case Report Form |
| **EDC** | Electronic Data Capture |
| **e-signature** | Electronic Signature |
| **FDA** | Food and Drug Administration |
| **GCP** | Good Clinical Practice |
| **HR** | Heart Rate |
| **ICH** | International Conference on Harmonisation |
| **ID** | Identification |
| **IND** | Investigational New Drug |
| **IRB** | Institutional Review Board |
| **ITT** | Intent to Treat |
| **IWRS** | Interactive Web Response System |
| **MedDRA** | Medical Dictionary for Regulatory Activities |

| | |
|---|---|
| **mITT** | Modified Intent to Treat |
| **NRS** | Numeric Rating Scale |
| **OLE** | Open-Label Extension |
| **PGIC** | Patient Global Impression of Change |
| **PHQ-9** | Patient Health Questionnaire-9 |
| **PI** | Package Insert |
| **PP** | Per Protocol |
| **RAP** | Reporting and Analysis Plan |
| **SAE** | Serious Adverse Event |
| **SCM** | Sternocleidomastoid |
| **TEAE** | Treatment Emergent Adverse Event |
| **TSQM** | Treatment Satisfaction Questionnaire for Medication |
| **TWSTRS** | Toronto Western Spasmodic Torticollis Rating Scale |
| **U** | Units |
| **USA** | United States of America |
| **USPI** | United States Prescribing Information |
| **WHO-Drug** | World Health Organisation Drug Dictionary |

# 2 INTRODUCTION

## 2.1 Disease Review

Focal dystonias are defined as abnormal co-contractions of agonist and antagonist muscles leading to torsion of body parts and abnormal postures. The dystonias are believed to be due to a neurochemical disturbance occurring within the basal ganglia. Cervical dystonia (CD) is the most common of the focal dystonias and is characterised by sustained involuntary contractions of the cervical muscles, leading to painful and disabling postures. Its onset may be acute and painful, or insidious and painless. Rotation of the chin (torticollis) and tilt (laterocollis), forward flexion (anterocollis), or backward extension of the head (retrocollis) can be present either in isolation or in various combinations. A prevalence of 89 per million of the population has been reported worldwide [1]. In five separate studies involving a total of 966 subjects [2,3,4,5,6], evaluation of the frequency of occurrence of the subtypes of CD showed the commonest type of deviation to be torticollis, with some degree of rotation noted in 82% [3] to 97% [2] of subjects. Laterocollis was the second most common deviation type observed, occurring in 42% [3] to 74% [6], while retrocollis occurred in 24% [6] to 38% [5] of subjects and anterocollis in 11% [6] to 30% [3]. Retrocollis rarely occurred in isolation (1%) [2,4] and pure anterocollis was never observed in these studies.

Spontaneous remission of CD occurs in about 15% of cases, but is frequently only temporary. The natural history in most cases is for progressive deterioration over the first five years, after which the disease stabilises. Spread to other segments of the body may also occur.

The available pharmacological treatments, including oral anticholinergics, benzodiazepines, L-dopa, haloperidol, and anticonvulsants are generally unsatisfactory. The effectiveness of surgical treatment remains uncertain [7]. Attempts at treatment using biofeedback, acupuncture and behaviour therapy have been unsatisfactory. Situational depression is common and may be helped by anti-depressant therapy. Analgesics and muscle relaxants may relieve the pain often associated with neck muscle spasms. Over the last 10 years botulinum toxins (BTX), injected intramuscularly into affected muscles, have shown promise for the treatment of CD [7] and four such toxins are approved for use in the United States of America (USA) and the European Union.

Botulinum toxin type A (BTX-A) is a potent neurotoxin isolated from the bacterium Clostridium botulinum, a gram-positive, spore-forming anaerobe. Botulinum toxin type A, a single-chain protein (molecular weight ~150,000 Dalton (Da)), is one of seven different serotypes (classed type A through G) of BTX produced by this organism. Proteins endogenous to the bacterium cleave the single-chain protein, resulting in a dichain neurotoxin containing a light chain (molecular weight ~50,000 Da) and a heavy chain (molecular weight ~100,000 Da) that remain linked by an inter-chain disulfide and non-covalent bonds.

Botulinum toxin type A acts selectively on peripheral cholinergic nerve endings, inhibiting acetylcholine release, effectively blocking signal transmission from nerve to muscle and inducing a temporary, partial chemodenervation of the injected muscle [8]. Injection into cervical muscles causes relaxation in muscle tone, fibre

atrophy and reduces motor unit action potential size [9], thereby decreasing the force of muscle contraction. The perceived benefits are relief from pain, increased range of free movement, improved resting posture and less overall disfigurement, leading to improved quality of life for subjects. Relief is only temporary, and the condition gradually returns as new neuromuscular junctions are created through collateral sprouting of affected axons, or with recovery of transmission of the native neuromuscular junction.

## 2.2 Compound Review

Dysport® 500 units (U) is a freeze dried preparation of Clostridium botulinum type A toxin-haemagglutinin complex (BTX-A-HAC) formulated with lactose (bulking agent) and human serum albumin. A more detailed description of the product is provided in Section 9.1. Per the United States Prescribing Information (USPI), Dysport is to be reconstituted with 1 mL of preservative free sodium chloride for injection (0.9%) to yield a solution containing 500 U of Dysport per mL.

The efficacy and safety of Dysport in the registered indications in the USA was established by two well-controlled clinical studies utilising a dosage range of 250 to 1000 U [10,11,12,13]. In addition, a Phase II dose ranging study investigated the efficacy and safety of 250, 500, and 1000 U of Dysport [14]. The subject population was restricted to naïve subjects, (previously untreated with BTX) and requiring injection of the sternocleidomastoid (SCM) and splenius capitis muscles due to predominantly rotational CD. The study demonstrated dose-response relationships for both efficacy and safety and concluded that, in this subject population, 500 U of Dysport was the optimal dose.

A Phase III, prospective, randomised, double-blind placebo-controlled investigation of the efficacy and safety of 500 U of Dysport for the treatment of CD was performed in Europe in 68 subjects. The study involved a single double-blind administration of Dysport or placebo. This study demonstrated safety and efficacy 4 and 8 weeks after treatment [15,16].

The most commonly reported adverse reactions, occurring in more than 5% of subjects who received 500 U of Dysport in the placebo-controlled clinical studies, in subjects with CD were muscular weakness, dysphagia (which is occasionally reported in the context of aspiration), dry mouth, injection site discomfort, fatigue, headache, neck pain, musculoskeletal pain, dysphonia, injection site pain, and eye disorders (consisting of blurred vision, diplopia, and reduced visual acuity and accommodation). Dysport should be administered with caution in subjects with pre-existing swallowing or breathing difficulties as these can worsen if the relevant muscles are affected. Aspiration is a particular risk when treating subjects with underlying respiratory problems. Other, less frequently reported, adverse reactions from the use of Dysport in this indication include injection site pain, influenza-like symptoms and dysphonia [17].

Further details can be found in the Investigator's Brochure [17].

## 2.3 Clinical Trial Rationale

The current USPI, based on the registration studies in CD mentioned above, allows for only one dilution of Dysport: 500 U in 1 mL volume. Feedback obtained from scientific experts and Investigators at medical advisory boards and in market

research data has pointed to the lack of scientific data supporting a 2 mL dilution as an obstacle to providing appropriate, safe and effective Dysport utilisation in the USA for subjects suffering from CD. Despite the lack of labelled information, the 2 mL dilution with Dysport reflects real world clinical practice in the USA [18].

The addition of data in the USPI supporting the safety and efficacy of a 2 mL dilution with Dysport will provide the clinician more flexibility in injection volume range to better equip them to meet the needs of a broader spectrum of subjects with CD.

Therefore, in this clinical study the majority of enrolled subjects will be previously treated with Botox® to reflect the real world clinical scenario in the USA.

# 3 STUDY OBJECTIVES

## 3.1 Primary Study Objective

The primary objective of the study is to evaluate the efficacy of Dysport 500 U/vial using 2 mL dilution compared with placebo for the treatment of CD.

## 3.2 Secondary Study Objectives

The secondary study objectives are to:

(1) Evaluate the safety of Dysport 500 U/vial using 2 mL dilution compared with placebo for the treatment of CD.
(2) Evaluate Investigator’s global assessment of improvement.
(3) Evaluate subject-related outcomes:
- Onset of symptom improvement.
- The effect on pain.
- The effect on changes from baseline in health-related quality of life.
- Satisfaction with treatment.

# 4 STUDY DESIGN

## 4.1 Overview

An overview of the study is presented in Figure 1.

**Figure 1 Study Flow Chart**

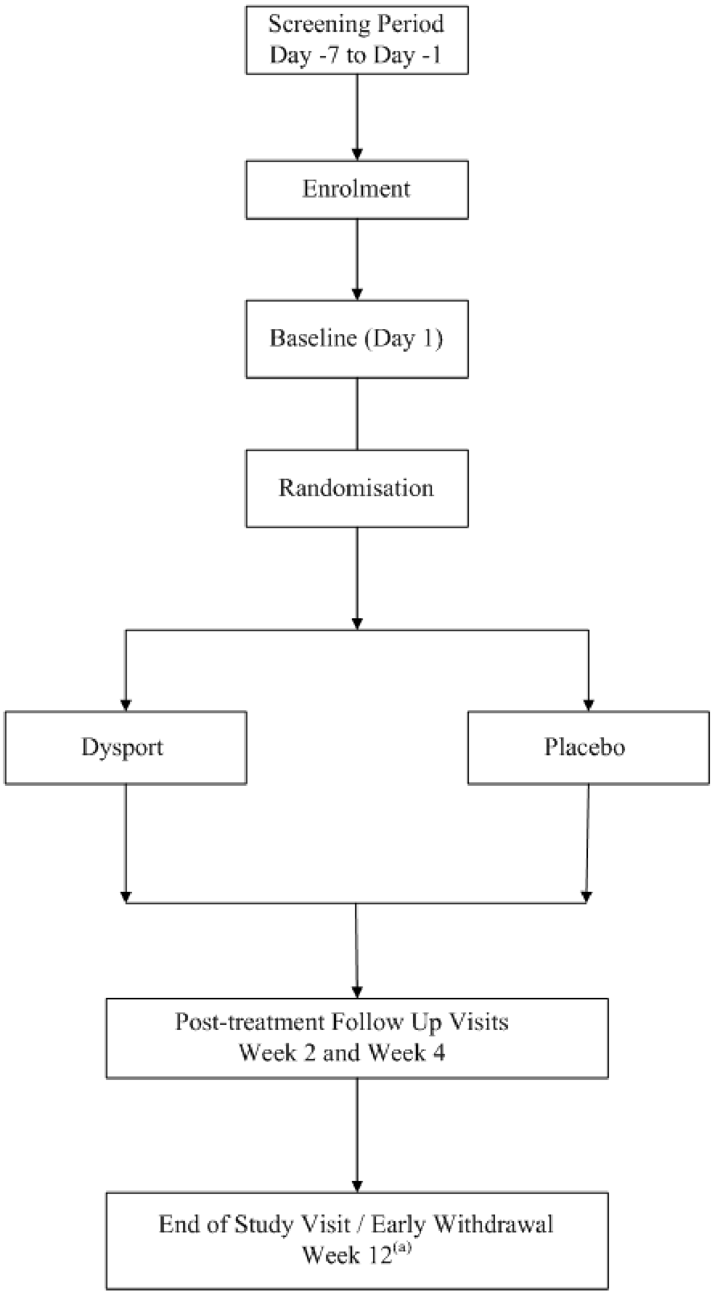


(a) Subjects who withdraw from the study early (i.e. before Week 12) will undergo all procedures required for the Week 12 visit.

### *4.1.1 Population Characteristics*

The study will include approximately 132 male and female subjects ≥18 years of age, who have a diagnosis of idiopathic CD with at least 18 months duration. These subjects may be BTX naïve or have received their last BTX treatment at least 12 weeks prior to study entry and having returned to their typical pre-treatment status. Additionally, subjects must have a Toronto Western Spasmodic Torticollis Rating Scale (TWSTRS)-total score ≥20 and TWSTRS-severity subscale score >10.

### *4.1.2 Design*

This is a phase IIIb, multicentre, randomised, double-blind, placebo-controlled study evaluating the efficacy and safety of Dysport 500 U/vial using a 2 mL dilution in adults with CD. In this study, the treatment dose of Dysport is between 250 U and 500 U. This study will be conducted at approximately 45 centres in the USA.

### *4.1.3 Structure*

All subjects are planned to have a single treatment in this study. All subjects who have had completed the Week 12 visit will be considered to have completed the study. The overall study duration will be approximately 13 months and will include subject recruitment (approximately 9 months) and last subject follow up (up to 4 months). Individual subject treatment duration will be between 4 and 16 weeks. All subjects who have had completed the Week 12 visit will be considered to have completed the study.

Subjects will be randomised in a ratio of 2:1 into the Dysport and placebo groups, respectively, and will be injected by intramuscular injection divided among a minimum of two clinically indicated muscles. Stratification will be performed according to BTX naïve or non-naïve status as assessed at baseline.

Study visits will include a screening visit, which will occur within 1 week prior to study treatment, a baseline treatment visit at Day 1 (this visit may occur on the same day as the screening visit at the discretion of the Investigator), and follow up visits at Weeks 2, 4 and 12 post-treatment or early withdrawal due to any reason.

All subjects who complete the Week 12 study visit will be offered entry into an open-label extension (OLE) study which consists of up to three treatment cycles of Dysport using the 2 mL dilution scheme. Between Weeks 4 and 8, subjects may be deemed eligible for early entry into the OLE study, i.e. before they reach the planned Week 12 study visit. In order to be eligible for early entry into the OLE study, subjects must fulfil the following criteria:

- At the scheduled Week 4 visit, or at an unscheduled visit occurring between Weeks 4 to 8, the TWSTRS total score as compared to baseline is reduced by ≤15%.
- Have no ongoing adverse events (AEs), which in the opinion of the Investigator are related to study treatment and that preclude the Subject from receiving continuing therapy.
- All other OLE study entry criteria.

#### *4.1.4 Stopping Rules and Discontinuation Criteria*

As this is a single treatment cycle study, subjects cannot have treatment discontinued after Day 1, although they may choose not to participate in the OLE study. However, subjects who withdraw informed consent to participate will be withdrawn from the study. Additionally, subjects will be withdrawn if in the Investigator's opinion withdrawal from the study is in the best interest of the subject.

The exact reason(s) for withdrawal must be recorded, if available. If possible a complete final examination, with all the Week 12 assessments, should be performed for all subjects who withdraw. In case of withdrawal due to an AE or pregnancy, the subject should be followed up by the Investigator outside the study framework.

#### *4.1.5 Early Study Termination*

The Sponsor may terminate this study at any time. Reasons for termination may include but are not limited to, the following:

- The incidence or severity of AEs in this or other studies point to a potential health hazard for study subjects.
- Insufficient subject enrolment.
- Any information becoming available during the study that substantially changes the expected benefit risk profile of the study treatments.
- Administrative reasons unrelated to study treatment or conduct.

### 4.2 Endpoints

#### *4.2.1 Efficacy Endpoints*

##### *4.2.1.1 Primary Efficacy Endpoint*

- Change from baseline in TWSTRS total score at the Week 4 visit.

##### *4.2.1.2 Secondary Efficacy Endpoints*

- Change from baseline in TWSTRS total score at the Week 2 visit.
- Clinical Global Impression of Change (CGIC) assessment of CD change at the Week 2 and Week 4 visits.
- Treatment response at the Week 2 and Week 4 visits. A treatment responder is defined as a subject who had at least a 30% reduction in the TWSTRS total score after treatment.
- Change from baseline in Cervical Dystonia Impact Profile (CDIP)-58 score at the Week 2 and Week 4 visits.

##### *4.2.1.3 Tertiary Efficacy Endpoints*

- Change from baseline in TWSTRS severity, disability and pain subscale scores at the Week 2, 4 and 12 visits.
- Change from baseline in TWSTRS total score at the Week 12 visit.
- Patient Global Impression of Change (PGIC) assessment of CD change at the Week 2, 4 and 12 visits.
- Change from baseline in pain Numeric Rating Scale (NRS) at the Week 2, 4 and 12 visits.

- Change from baseline in Brief Pain Inventory (BPI) short form at the Week 2, 4 and 12 visits.
- Change from baseline in Patient Health Questionnaire (PHQ)-9 at the Week 2, 4 and 12 visits.
- Modified Treatment Satisfaction Questionnaire for Medication (TSQM)-9 change at the Week 2, 4 and 12 visits.
- Clinical Global Impression of Change assessment of CD change at the Week 12 visit.
- Treatment response at the Week 12 visit.
- Change from baseline in CDIP-58 score at the Week 12 visit.

#### *4.2.2 Safety Endpoints*

- Treatment emergent AEs (TEAEs) (including information on seriousness, intensity, drug relationship and TEAEs leading to study withdrawal) from the granting of informed consent and at each study visit.
- Suicidal ideation and behaviour will be assessed using the Columbia-Suicide Severity Rating Scale (C-SSRS) at each study visit.
- Absolute values and changes from baseline in vital signs and physical examination findings at screening and at the end of study or early withdrawal visits.

### 4.3 Justification of Design

The current USPI, based on the registration studies in CD mentioned above, allows for only one dilution: 500 U in 1 mL volume. Feedback obtained from scientific experts and Investigators at medical advisory boards and in market research data has pointed to the lack of scientific data supporting a 2 mL dilution as an obstacle to providing appropriate, safe and effective Dysport utilisation in the USA for subjects suffering from CD. Despite the lack of labelled information, the 2 mL dilution with Dysport reflects real world clinical practice in the USA [18].

The addition of data in the USPI supporting the safety and efficacy of a 2 mL dilution with Dysport will provide the clinician more flexibility in injection volume range to better equip them to meet the needs of a broader spectrum of subjects with CD.

Therefore, in this clinical study the majority of enrolled subjects will be previously treated with Botox to reflect the real world clinical scenario in the USA.

#### *4.3.1 Study Population for Analysis*

Subjects to be included for analysis will include approximately 132 male and female subjects who are ≥18 years of age.

The modified intent to treat (mITT) population will be the population of primary interest for the efficacy analyses.

#### *4.3.2 Study Duration*

The overall study duration will be approximately 13 months and will include subject recruitment (approximately 9 months). The study will be considered to have started when the first subject has provided signed informed consent. The study will be considered to have finished after the last subject has completed the last follow up visit in the study.

For each individual subject, study participation will be between 4 and 16 weeks. Study visits will include a screening visit, which will occur within 1 week prior to study treatment, a baseline treatment visit at Day 1 (this visit may occur on the same day as the screening visit at the discretion of the Investigator), and follow up visits at Weeks 2, 4 and 12 post-treatment or early withdrawal due to any reason. The subjects' participation in the study is considered to have ended at the time of the last visit (Week 12) or early withdrawal from the study.

# 5 COMPLIANCE WITH GOOD CLINICAL PRACTICE, ETHICAL CONSIDERATIONS & INFORMED CONSENT

## 5.1 Compliance with Good Clinical Practice and Ethical Considerations

This study must be conducted in compliance with institutional review boards (IRBs), informed consent regulations, the Declaration of Helsinki and International Conference on Harmonisation (ICH) Good Clinical Practice (GCP) Guidelines [19].

For Electronic Data Capture (EDC) studies the following regulations must be included: Food and Drug Administration (FDA), 21 CFR Part 11, Electronic Records, Electronic Signatures, and FDA, Guidance for Industry: Computerized Systems Used in Clinical Trials [20,21].

In addition, this study will adhere to all USA FDA regulatory requirements and relevant company policies.

Before initiating a study, the Investigator/institution should have written and dated approval/favourable opinion from the IRB for the study protocol/amendment(s), written informed consent form, any consent form updates, patient emergency study contact cards, subject recruitment procedures (e.g. advertisements), any written information to be provided to subjects and a statement from the IRB that they comply with GCP requirements. The IRB approval must identify the protocol version as well as the documents reviewed.

After IRB approval, changes will require a formal amendment. Once the study has started, amendments should be made only in exceptional circumstances. Changes that do not affect subject safety or data integrity are classified as administrative changes and generally do not require IRB approval. If more significant changes to the protocol are concerned, the IRB must be informed and, if necessary, approval sought prior to implementation. Approval on administrative changes will be obtained if required by local/site IRB.

## 5.2 Informed Consent

Prior to study entry, the Investigator, or a person designated by the Investigator, will explain the nature, purpose, benefits and risks of participation in the study to each subject, subject's legally acceptable representative or impartial witness. Written informed consent must be obtained prior to the subject entering the study (before initiation of any study-related procedure and administration of the study treatment). Sufficient time will be allowed to discuss any questions raised by the subject.

The Sponsor will provide a sample informed consent form. The final version controlled form must be agreed to by the Sponsor, and the IRB and must contain all elements included in the sample form, in language readily understood by the

subject. Each subject's original consent form, personally signed and dated by the subject or by the subject's legally acceptable representative, and by the person who conducted the informed consent discussion, will be retained by the Investigator. The Investigator will supply all enrolled subjects with a copy of their signed informed consent.

The consent form may need to be revised during the study should important new information become available that may be relevant to the safety of the subject or as a result of protocol amendments. In this instance approval should always be given by the IRB. It is the Investigator's responsibility to ensure that all subjects subsequently entered into the study and those currently in the study sign the amended form. This is documented in the same way as previously described. Subjects who have completed the study should be informed of any new information that may impact on their welfare/wellbeing.

The Investigator should, with the consent of the subject, inform the subject's primary physician about their participation in the clinical study.

# 6 STUDY POPULATION

## 6.1 Screening Log and Number of Subjects

Each Investigator will maintain a record of all subjects who were considered eligible for entry into the study but who were not enrolled. For each subject, the primary reason for exclusion will be recorded.

Each Investigator will also maintain a record of all subjects enrolled into the study (i.e. who signed the informed consent form). In the event that the subject was not receiving study treatment, the primary reason will be recorded.

It is planned to recruit approximately 132 subjects at approximately 45 centres in the USA. Section 11.2 provides a discussion of sample size.

## 6.2 Inclusion Criteria

All subjects must fulfil the following:

(1) Written informed consent prior to any study related procedures.

(2) Male or female subjects ≥18 years of age.

(3) Primary diagnosis of idiopathic CD at least 9 months since onset, and either:
- Previously untreated with BTX (naïve to BTX) or
- Currently treated with Botox at a total dosing range of 100 to 200 U and ≤60 U in the SCM muscle at the last injection cycle, and having had a satisfactory treatment response in the enrolling Investigator's judgment during the last two sequential cycles of Botox within the past 18 months for the treatment of CD.

(4) For non-naïve BTX subjects, a minimum interval of 12 weeks since the last Botox injection and having returned at least to their typical pre-treatment status as assessed by the Investigator.

(5) Non-naïve subjects may have received any other formulations of BTX prior to study entry as long as they have had a satisfactory clinical response to the last two injection sessions that must have been Botox.

(6) TWSTRS meeting the following criteria at baseline:

 (a) TWSTRS-total score ≥20.

(b) TWSTRS-severity subscale score >10.

## 6.3 Exclusion Criteria

Subjects will be excluded if any of the following apply:

(1) In apparent remission from CD.
(2) Diagnosis of pure retrocollis or pure anterocollis.
(3) For non-naïve subjects, previous poor response (as determined by standard practice) to either of the last two Botox treatments for CD.
(4) Known requirement of <100 U or >200 U Botox injected into the neck muscles.
(5) Known resistance to any BTX-A.
(6) Lack of therapeutic response to previous Dysport treatments for CD.
(7) Requirement for BTX injection to site(s) for disorders other than CD and unable to avoid such treatment(s) for the duration of the study.
(8) Known hypersensitivity to BTX or related compounds, or any component in the study drug formulation.
(9) Allergy to cow's milk protein.
(10) Myasthenia gravis, other disease of the neuromuscular junction or clinically significant, persistent neuromuscular weakness, or disease or symptoms that can interfere with the TWSTRS scoring.
(11) Total body weight <95 lbs (43.09 kg).
(12) Previous phenol injections to the neck muscles.
(13) Previous myotomy or denervation surgery involving the neck or shoulder region or deep brain stimulation to treat CD.
(14) Cervical contracture that limits passive range of motion.
(15) Physiotherapy initiated less than 4 weeks before study entry or expected to be initiated during the study.
(16) Treatment with aminoglycoside antibiotics within the last 30 days prior to study treatment.
(17) Current or expected requirement for concomitant medication that may interfere with the evaluation of study treatment (note: muscle relaxants, narcotics, and benzodiazepines are permitted if the dosage has been stable for the 4 weeks prior to study treatment and is expected to remain at this dose until the Week 4 assessment. Every effort should be made to keep concomitant CD treatment constant throughout the study. However, changes in pain medication are acceptable if absolutely necessary according to clinical judgment).
(18) Received any investigational new drug or device within 30 days prior to inclusion in the study.
(19) Pregnant and/or lactating females.
(20) Females of childbearing potential with a positive pre-study urine pregnancy test (a positive urine pregnancy test can be confirmed by serum pregnancy test at the discretion of the Investigator) and subjects, or their partners, who do not agree to use adequate contraception (hormonal or barrier method of birth control) prior to injection of study treatment and for the duration of study participation. Non-childbearing potential is defined as post-menopause

for at least 1 year, surgical sterilisation at least 3 months before entering screening, or hysterectomy.

(21) Individuals with family or employee relationship to investigative site staff or Sponsor staff involved in the conduct of the study.

(22) Any medical condition that compromises compliance with the objectives and procedures of this protocol or precludes the administration of BTX, including swallowing and other respiratory abnormality, as judged by the Investigator.

(23) In the opinion of the Investigator the subject is unable and/or unwilling to comply fully with the protocol and the study instructions.

### 6.4 Subject Withdrawal Criteria

As this is a single treatment cycle study, discontinuation from study treatment is not applicable.

Under no circumstances will subjects be enrolled more than once. If one or more of the following occurs, the subject will be withdrawn from the study:

- Withdrawal of informed consent.
- Investigator and/or Sponsor's decision to withdraw the subject if it is considered to be in the subject's best interest.
- Continuous failure to comply with the provisions of the study protocol which is likely to have an adverse impact on the safety or well-being of the subject, or could jeopardise the scientific value of the study.

The subject's participation in the study is voluntary and the subject may refuse to participate in or withdraw from the study, at any time, without penalty or loss of benefits to which the subject is otherwise entitled.

### 6.5 Discontinuation/Withdrawal Procedures

If the subject is withdrawn from the study (i.e. ceases participation in the study prior to completion of the assessments planned in the protocol), the primary reason should be recorded in the electronic case report form (eCRF). Withdrawal due to AEs should be distinguished from withdrawal due to insufficient response.

The Investigator will provide or arrange for appropriate follow up (if required) for subjects withdrawing from the study, and will document the course of the subject's condition. Where the subject has withdrawn due to an AE the Investigator should follow the procedures documented in Section 10 in order to assess the safety of the study treatment.

## 7 METHODOLOGY

### 7.1 Study Schedule

The schedule of observations and assessments during the study are summarised in Table 1.

**Table 1 Schedule of Assessments**

| Study Period | Screening | Baseline | Post-treatment Follow up | | End of Study /Early Withdrawal[b] |
|---|---|---|---|---|---|
| **Week** | | | **W2** | **W4** | **W12** |
| **Day/Week** | **D-7 to D-1** | **D1** | **D14+2 Days** | **D28 ±2 Days** | **D84+28 Days** |
| **Visit** | **V1[a]** | **V2** | **V3** | **V4** | **V5** |
| Written informed consent | X | | | | |
| Eligibility criteria | X | | | | |
| Demographic data[c] | X | | | | |
| Cervical dystonia history | X | | | | |
| Medical/surgical history | X | | | | |
| BTX history | X | | | | |
| Physical examination | X | | | | X |
| Urine pregnancy test | X[d] | | | | X |
| Prior CD treatment and medications[e] | X | | | | |
| Vital signs[f] | X | | | | X |
| Height and weight | X | | | | |
| Blood biochemistry | X | | | | X |
| TWSTRS total and subscale scores | | X | X | X | X |
| Clinical Global Impression of Change (CGIC) | | | X | X | X |
| Subject's CDIP-58 score | | X | X | X | X |
| Patient Global Impression of Change (PGIC) | | | X | X | X |
| Pain NRS | | X | X | X | X |
| Brief Pain Inventory (BPI) short form | | X | X | X | X |
| Patient Health Questionnaire (PHQ)-9 | | X | X | X | X |
| Modified treatment satisfaction questionnaire for medication (TSQM-9) | | | X | X | X |
| Columbia-Suicide Severity Rating Scale (C-SSRS) | | X | X | X | X |
| Adverse events | X | X | X | X | X |
| Concomitant medications/therapies[e] | | X | X | X | X |
| Randomisation | | X | | | |
| Study treatment administration | | X | | | |

Key: V=visit, D=day; W=week, BTX=botulinum toxin, CD=cervical dystonia, TWSTRS=Toronto Western Spasmodic Torticollis Rating Scale, CDIP-58=Cervical Dystonia Impact Profile-58, Pain NRS=pain numeric rating scale.

(a) If appropriate, the screening visit can be the same day as baseline.

(b) Subjects who withdraw from the study early (i.e. before Week 12) will undergo all procedures required for the end of study/early withdrawal visit.

(c) Demography including sex, date of birth/age, ethnicity and race.

(d) A urine pregnancy test will be performed for all female subjects of childbearing potential. A positive urine pregnancy test can be confirmed by serum pregnancy test at the discretion of the Investigator. If this is found to be positive, the subject will be excluded from the study.

(e) Prior medications will be any medications administered within 4 months before study start but stopped more than 30 days before the screening visit. Concomitant medications/ treatments will be all medications administered within 30 days before the screening visit.

(f) Sitting blood pressure, heart rate, oral temperature and respiratory rate.

## 7.2 Study Visits

### *7.2.1 Pre-study Screening (Day -7 to Day -1, Visit 1)*

Screening will be conducted between Day -7 to Day -1. If appropriate, the screening visit can be the same day as baseline.

Written informed consent should be obtained prior to enrolment when the following assessments will be performed:

- Eligibility check (inclusion/exclusion criteria).
- Demographics (sex, date of birth/age, ethnicity and race).
- Medical/surgical history, including ongoing medical history.
- Cervical dystonia history.
- Botulinum toxin history.
- Physical examination.
- Prior CD treatment and medications/therapies. Prior medications will be any medications administered within 4 months before study start but stopped more than 30 days before the screening visit. Concomitant medications/ treatments will be all medications administered within 30 days before the screening visit.
- Vital signs (sitting blood pressure (BP), heart rate (HR), oral temperature and respiratory rate).
- Height and weight.
- Blood sampling for biochemistry.
- Urine pregnancy test for all female subjects of childbearing potential. If this is found to be positive, the subject will be excluded from the study.
- Adverse events.

### *7.2.2 Baseline (Day 1, Visit 2)*

The following procedures will be performed prior to each subject receiving study treatment:

- Toronto Western Spasmodic Torticollis Rating Scale total and subscale scores.
- Subject's CDIP-58 score.
- Pain NRS.
- Brief Pain Inventory short form.
- Patient Health Questionnaire-9.
- Columbia-Suicide Severity Rating Scale.
- Concomitant medications.
- Adverse events.
- Randomisation.
- Study treatment administration.

### *7.2.3 Post-treatment Follow Up (Week 2±2 days, Visit 3 and Week 4±2 days, Visit 4)*

The following assessments should be performed at the Week 2 and Week 4 follow up visits for all subjects:

- Toronto Western Spasmodic Torticollis Rating Scale total and subscale scores.
- Clinical Global Impression of Change.

- Subject's CDIP-58 score.
- Patient Global Impression of Change.
- Pain NRS.
- Brief Pain Inventory short form.
- Patient Health Questionnaire-9.
- Modified TSQM-9.
- Columbia-Suicide Severity Rating Scale.
- Concomitant medications.
- Adverse events.

#### *7.2.4 Study Completion or Early Withdrawal (Week 12+28 days, Visit 5)*

The following assessments should be performed at the study completion or early withdrawal visit:

- Vital signs (sitting BP, HR, oral temperature and respiratory rate).
- Blood sampling for biochemistry.
- Urine pregnancy test for all female subjects of childbearing potential. A confirmatory serum test to be performed if urine pregnancy test is positive.
- Brief physical examination.
- Toronto Western Spasmodic Torticollis Rating Scale total and subscale scores.
- Clinical Global Impression of Change.
- Subject's CDIP-58 score.
- Patient Global Impression of Change.
- Pain NRS.
- Brief Pain Inventory short form.
- Patient Health Questionnaire-9.
- Modified TSQM-9.
- Columbia-Suicide Severity Rating Scale.
- Concomitant medications.
- Adverse events.

# 8 STUDY EVALUATIONS

For the timing of assessments during the study, refer to the study schedule in Section 7.1.

## 8.1 Efficacy Endpoints and Evaluations

Efficacy assessments will include TWSTRS total and subscale (severity, disability and pain) scores, subject's assessment of pain, Investigator's and subject's global assessment of change of CD, quality of life questionnaires and subject's overall satisfaction with treatment.

### *8.1.1 Primary Efficacy Endpoint and Evaluation*

TWSTRS is an assessment scale used to measure the impact of CD on subjects. It is comprised of three subscales: severity, disability and pain, each of which is scored independently [6]. The total score from these three subscales comprises the TWSTRS total score which can have a value from 0–87 (best to worst). Further details are provided in Appendix 1.

The TWSTRS total score will be used to assess the degree of CD. It will be assessed by the Investigator prior to the study treatment at baseline (Day 1) and at all post-treatment visits. The primary efficacy endpoint will be the change from baseline in TWSTRS total score at the Week 4 visit.

### 8.1.2 Secondary Efficacy Endpoints and Evaluations

#### 8.1.2.1 Toronto Western Spasmodic Torticollis Rating Scale

The change from baseline in TWSTRS total score, as described in Section 8.1.1, will be assessed by the Investigator at the Week 2 visit as a secondary endpoint.

In addition, the treatment response at the Week 2 and Week 4 visits will be analysed as secondary endpoints. A treatment responder is defined as a subject who had at least a 30% reduction in the TWSTRS total score after treatment.

#### 8.1.2.2 Clinical Global Impression of Change

The CGIC is an Investigator-reported assessment of the global clinical change in CD since study treatment administration. The CGIC will be assessed by the Investigator using a 7-point Likert scale ranging from +3 (very much improved) to -3 (very much worse). Further details are provided in Appendix 2.

The CGIC will be assessed by the Investigator at the Week 2 and Week 4 visits as secondary endpoints.

#### 8.1.2.3 Cervical Dystonia Impact Profile-58

The CDIP-58 scale is a subject-based rating scale measuring the health impact in CD measured in eight health dimensions: head and neck symptoms, pain and discomfort, upper limb activities, walking, sleep, annoyance, mood, and psychosocial functioning [22]. Further details are provided in Appendix 3.

The change from baseline in CDIP-58 score at the Week 2 and Week 4 visits will be analysed as secondary endpoints.

### 8.1.3 Tertiary Efficacy Endpoints and Evaluations

#### 8.1.3.1 Toronto Western Spasmodic Torticollis Rating Scale

The change from baseline in TWSTRS total score, as described in Section 8.1.1, and the treatment response, as described in Section 8.1.2.1, will be assessed by the Investigator at the Week 12 visit as tertiary endpoints.

The TWSTRS severity, disability and pain subscales will also be used to assess the degree of CD. The change from baseline in TWSTRS subscale scores will be assessed by the Investigator at the Week 2, 4 and 12 visits as a tertiary endpoint.

#### 8.1.3.2 Patient Global Impression of Change

The PGIC is a subject-reported assessment of the subject's perception of the change in their CD since study treatment administration. The PGIC will be assessed by the subject using a 7-point Likert scale ranging from +3 (very much improved) to -3 (very much worse). Further details are provided in Appendix 4.

The PGIC will be assessed by the subject at the Week 2, 4 and 12 visits as a tertiary endpoint.

#### 8.1.3.3 *Numeric Rating Scale: Subject Assessment of Pain*

Subjects will be asked to rate their pain in the previous 24 hours using the pain NRS. The NRS ranges from 0 (no pain) to 10 (worst pain possible). Further details are provided in Appendix 5.

The NRS will be assessed by the subject at baseline and at the Week 2, 4 and 12 visits. The change from baseline will be analysed as a tertiary endpoint.

#### 8.1.3.4 *Brief Pain Inventory*

Subjects will be asked to rate the impact of their CD pain on seven areas in the past 2 weeks using the BPI short form. The BPI short form ranges from 0 (does not interfere) to 10 (completely interferes). Further details are provided in Appendix 6.

The subject will assess his/her pain using the BPI short form at baseline and at the Week 2, 4 and 12 visits. The change from baseline will be analysed as a tertiary endpoint.

#### 8.1.3.5 *Patient Health Questionnaire*

The PHQ-9 is a general measure of depression and ranges from 0 (not at all) to 3 (nearly every day). Subjects will be asked to answer nine questions relating to the past 2 weeks. Further details are provided in Appendix 7.

The subject will assess his/her depression using the PHQ-9 at baseline and at the Week 2, 4 and 12 visits. The change from baseline will be analysed as a tertiary endpoint.

#### 8.1.3.6 *Modified Treatment Satisfaction Questionnaire for Medication*

The TSQM-9 is a general measure of treatment satisfaction used to evaluate change in the subjects global satisfaction after treatment. The modified TSQM-9 includes six items across four domains. Further details are provided in Appendix 8. Subjects will be asked to complete the modified TSQM-9 questionnaire at the Week 2, 4 and 12 visits. Treatment satisfaction will be assessed as a tertiary endpoint.

#### 8.1.3.7 *Clinical Global Impression of Change*

The CGIC, as described in Section 8.1.2.2, will be assessed by the Investigator at the Week 12 visit and the change from baseline will be analysed as a tertiary endpoint.

#### 8.1.3.8 *Cervical Dystonia Impact Profile-58*

The CDIP-58, as described in Section 8.1.2.3, will be assessed by the Investigator at the Week 12 visit as a tertiary endpoint.

## 8.2 Safety Endpoints and Evaluations

- Treatment emergent AEs (TEAEs) (including information on seriousness, intensity, drug relationship and TEAEs leading to study withdrawal) from the granting of informed consent and at each study visit.
- Suicidal ideation and behaviour will be assessed using the C-SSRS at each study visit.
- Absolute values and changes from baseline in vital signs and physical examination findings at screening and at the end of study or early withdrawal visits.

### *8.2.1 Adverse Events*

Adverse events will be monitored from the time that the subject gives informed consent to the time when the subject's participation in the study is considered to have ended (as defined in Section 4.3.2). Adverse events will be elicited by direct, non-leading questioning or by spontaneous reports. Further details for AE reporting can be found in Section 10.

### *8.2.2 Physical Examination*

A physical examination will be carried out by a physician. If in the opinion of the Investigator there are any clinically significant changes in the physical examination findings (abnormalities) they will be recorded as AEs.

### *8.2.3 Vital Signs*

Sitting BP, HR, oral temperature and respiratory rate will be recorded at screening and at the end of the study or early withdrawal. Height and weight will be measured at screening.

### *8.2.4 Electrocardiogram*

No electrocardiograms will be performed in this study.

### *8.2.5 Clinical Laboratory Tests*

Blood samples for clinical laboratory testing (biochemistry only) will be taken at screening and at the end of study or early withdrawal visit and will consist of the following:

**Biochemistry** - urea, creatinine, total bilirubin, conjugated bilirubin, chloride, bicarbonate, sodium, potassium, calcium, inorganic phosphate, alkaline phosphatase, aspartate aminotransferase, alanine aminotransferase, gammaglutamyl transferase, albumin, total protein, total cholesterol, triglycerides, and glucose.

**Pregnancy** A urine sample will be collected for a pregnancy test at screening for female subjects of childbearing potential. A positive urine pregnancy test can be confirmed by a serum pregnancy test at the discretion of the Investigator. If this is found to be positive, the subject will be excluded from the study. A urine sample will also be collected for a pregnancy test at the end of study/early withdrawal visit. At this visit, a confirmatory serum test will be performed if the urine pregnancy test is positive.

Clinical laboratory tests will be performed by a central laboratory. Details of the methodology and reference ranges will be outlined in the study manual and provided in the Trial Master File.

Laboratory test results will be recorded by the central laboratory and provided to the study Sponsor and to the study sites for evaluation of clinically significant changes. All clinically relevant abnormal laboratory tests occurring during the study will be repeated at appropriate intervals until they return to baseline or to a level deemed acceptable by the Investigator and the Sponsor's clinical monitor (or his/her designated representative), or until the abnormality is explained by an appropriate diagnosis. See Section 10.2.4 for abnormal laboratory tests that should be recorded as AEs in the eCRF.

#### *8.2.6 Suicidality*

To further assess risks, the C-SSRS has been added to the study. This is required for use in all clinical studies involving any drug being developed for any psychiatric indication, as well as for all antiepileptic drugs and other neurologic drugs with central nervous system activity, or the potential thereof, both in-patient and out-patient, including multiple dose phase I studies involving healthy subjects.

Suicidal ideation and behaviour will be assessed with the C-SSRS at baseline and at each subsequent visit. The C-SSRS is an Investigator administered measure of suicidal behaviour devised to detect potential suicidal thoughts or behaviours during a clinical study. It consists of a Baseline form that assesses lifetime suicidal ideation, ideation intensity and behaviour, and a form for assessing current suicidal ideation and behaviour. The C-SSRS consists of a series of questions, and can be administered during face to face interview or over the telephone. The Investigators can also administer the C-SSRS at any additional time points if there is reason to suspect possible suicidal risk. The C-SSRS will permit assessment of suicidal ideation and behaviour over the course of the study [23]. Three C-SSRS forms will be used in this study: one for already enrolled subjects, one for baseline assessment, and one for assessments since last visit. Further details are provided in Appendix 9, Appendix 10 and Appendix 11.

### 8.3 Total Blood Volume

The scheduled total volume of blood taken during the course of the entire study (for biochemistry only) will not exceed 12.0 mL.

### 8.4 Pharmacodynamic Endpoints and Evaluations

There are no pharmacodynamic evaluations in this study.

### 8.5 Pharmacokinetic Endpoints and Evaluations

There are no pharmacokinetic evaluations in this study.

### 8.6 Blood Samples for Putative Antibodies

There will be no testing for the presence of antibodies to BTX-A in this study.

## 9 STUDY TREATMENTS

### 9.1 Study Treatments Administered

It is forbidden to use study treatment for purposes other than as defined in this protocol. Administration of the study treatment will be administered by the Investigator.

#### *9.1.1 Dysport*

Dysport is provided in Type I, 3.0 mL glass vials containing 500 U (nominal) of BTX-A-HAC as a white lyophilised powder for reconstitution.

| **Active Constituent:** | **Per Vial** |
|---|---|
| Clostridium BTX-A-HAC | 500 U* |
| **Other Constituents:** | |
| Human serum albumin | 125 µg |
| Lactose monohydrate | 2.5 mg |

*One unit (U) is defined as the median lethal intraperitoneal dose in mice.

All vials are closed by 13 mm freeze drying closures oversealed by 13 mm aluminium overseals with a centre hole, crimped over.

Dysport drug product should be stored at the recommended temperature (between 2ºC and 8ºC).

The product does not contain any antimicrobial agent. Therefore, the product should be used within 24 hours after reconstitution. Dysport should not be frozen. Protect from light.

### *9.1.2 Placebo*

Placebo is provided in Type I, 3.0 mL glass vials and will be undistinguishable from the active product. The placebo will contain only the excipients described in Dysport, without the addition of toxin, as a white lyophilised powder for reconstitution.

| **Other Constituents:** | **Per Vial** |
|---|---|
| Human serum albumin | 125 µg |
| Lactose monohydrate | 2.5 mg |

All vials are closed by 13 mm freeze drying closures oversealed by 13 mm aluminium overseals with a centre hole, crimped over.

Placebo product should be stored at the recommended temperature (between 2ºC and 8ºC).

The product does not contain any antimicrobial agent. Therefore, the product should be used within 24 hours after reconstitution. Placebo should not be frozen. Protect from light.

### *9.1.3 Administration Procedures*

At study entry, subjects will be randomised 2:1 into one of two study treatment groups (Dysport or placebo). All eligible subjects will receive either Dysport or placebo by intramuscular injection. The dose of Dysport will be between 250 and 500 U.

Subjects randomised to the Dysport group and who are naïve to BTX treatment for CD will receive 500 U of Dysport in a minimum of two clinically affected neck muscles. The sites of injection and the dose per site will be determined by the Investigator according to the standard practice and disease presentation. Non-naïve subjects who are randomised to the Dysport group will receive a dose up to 500 U of Dysport and based on the ratio of 2.5:1 (Dysport:Botox from previous treatment dose for CD). Subjects in this group will receive Dysport injected into the same neck muscles that were injected during the last two sequential cycles of Botox within the past 18 months for the treatment of CD. The amount of study treatment injected into the SCM muscle should be limited to ≤0.6 mL (150 U Dysport). Electromyography may be used to identify the sites of injection for any subjects.

Before administration, the Dysport and placebo vials will be reconstituted at the investigational site with 2 mL preservative free sodium chloride for injection (0.9%). Detailed instructions will be provided for the volume which needs to be withdrawn from the reconstituted Dysport vials.

All of the boxes of study treatment will be identical in appearance allowing the double-blind conditions of the study to be maintained.

## 9.2 Subject Identification and Allocation to Study Treatment

All subjects enrolled must be identifiable throughout the study. The Investigator will maintain a list of subject numbers and names to enable records to be found at a later date if required.

At screening, potential subjects will be allocated a subject number. Following confirmation of eligibility for the study, subjects will be given a randomisation/treatment allocation number and allocated to one of the treatment groups specified in Section 9.1.

Approximately 132 subjects will be randomised in this study.

### *9.2.1 Randomisation*

The Sponsor's Randomisation Manager, who is a statistician independent from the study, will prepare two lists which will be performed in blocks and will be based on computer-generated randomisation list.

- List A: a list of randomisation numbers stratified for subjects who are BTX treatment naïve or non-naïve at baseline will be generated with a 2:1 ratio Dysport:placebo.
- List B: a list of treatment numbers, which will be specified on the treatment packs, to be dispatched to the sites in order to dispense the drug. This list will be produced on a 1:1 basis (1 Dysport:1 placebo).

The randomisation, as well as the treatment number assignation at the drug dispensation, will be managed by an Interactive Web response System (IWRS) service.

After eligibility is confirmed, subjects will be randomised at baseline, in sequential order within each centre (and within each level of strata).

Subjects meeting the randomisation criteria will be assigned to a randomisation number and will be allocated to the associated treatment arm, by the IWRS. An appropriate treatment number will be also allocated by the IWRS according to the allocated treatment group. The IWRS will also manage all the logistical aspects of treatments (e.g. drug resupplies and expiry dates).

This service provides Investigators, site co-ordinators and project team members with a 24-hour per day, 7-day per week service (additional details may be found in the IWRS reference manual provided to each site). In case of medical or technical randomisation or dispensation queries, a 24-hour helpline is available - see supporting information in the Investigator Site File.

Recruitment will stop once approximately 132 subjects have been randomised. Randomised subjects who terminate their study participation for any reason before administration of the randomised study treatment will retain their randomisation and treatment numbers (i.e. the treatment number will not be reused). The next subject is given another randomisation number and another treatment number even if he should receive the same treatment. Subjects who leave the study early will not be replaced.

The Sponsor's Randomisation Manager will keep the master lists. A copy of the list of treatment numbers (list B) will be confidentially supplied to the Clinical Trial Supplies Unit (CTSU), Beaufour Ipsen Industrie, Rue d'Ethe Virton, 28100 Dreux, France and to the CRO in charge of IWRS. Similarly, a copy of the list of

randomisation numbers stratified for patients who are BTX treatment naïve or non-naïve at baseline (list A) will be also confidentially sent to the CRO in charge of IWRS. The master list and the copies supplied to the CTSU and the CRO in charge IWRS will be kept confidential in a secure location. Access to the randomisation lists must be restricted until authorisation is given to unblind for analysis.

#### *9.2.2 Blinding, Emergency Envelopes and Breaking the Blind*

All of the study treatment will be similar in size, colour, smell, taste and appearance allowing the blinded conditions of the study to be maintained.

One set of individual sealed code-break envelopes will be prepared by the Sponsor's Randomisation Manager to enable emergency code-break procedures of individual subjects without compromising the blind of the study. This set will be provided to the Central Department of Pharmacovigilance at Ipsen.

In the event of a serious adverse event (SAE) or unexpected AE, which requires the identification of the study treatment group, the Investigator should first contact the Pharmacovigilance Emergency Contact as shown on the cover page of the protocol. The Investigator should then review the case status and all pertinent information with the Emergency Contact who will organise a break of blind as required. Where necessary the Investigator may break the blind by asking the IWRS to obtain the subjects treatment identification. In addition, the set of sealed code-break envelopes will be held by Central Department of Pharmacovigilance at Ipsen in case of IWRS failure.

The date and reason for identifying the treatment group will be recorded in the eCRF.

### 9.3 Study Treatment Supply, Packaging and Labelling

The study treatment will be packaged by CTSU, Beaufour Ipsen Industrie, Rue d'Ethe Virton, 28100 Dreux, France and delivered to the interim storage facility based in the USA. Then the interim storage facility will send the treatment kits to each investigational site. A sufficient quantity of study treatment will be supplied as well as an acknowledgement of receipt form.

The Sponsor's representative will receive a Certificate of Analysis for which batch of study treatment has been used under their study and the Pack Batch Release Certificate which reflects the product release. The core label texts for all packaging units will be translated or adjusted, to be in compliance with applicable regulatory requirements, national laws in force and in accordance with the local languages. A description of the core text of the study treatment labels is displayed below:

- Sponsor name, address and telephone number
- Study Number
- Product Name (Dysport 500U or placebo)
- Pharmaceutical dosage form
- Route of administration
- Quantity of dose units
- Batch number
- Treatment number

- Specific blank spaces to enter the subject identification and randomisation number
- "Caution: new drug limited by Federal Law to investigational use"
- Storage conditions
- Expiry date

The Investigator, or designee, will only dispense study treatment to subjects included in this study. Each subject will only be given the study treatment carrying his/her number. The dispensing for each subject will be documented in the eCRF.

## 9.4 Compliance

Study treatment will be administered intramuscularly at the study centre by the Investigator, thus, subject compliance with treatment is not expected to be an issue. Drug accountability records will be maintained by the Investigator documenting that subject received allocated drug.

## 9.5 Study Treatment Storage and Accountability

The Investigator, or an approved representative (e.g. pharmacist), will ensure that all study treatment is stored in a secured area, under recommended temperature monitored storage conditions, in accordance with applicable regulatory requirements and will be reconstituted and dispensed by qualified staff members.

Used vials, which should not be emptied, must be placed in suitable containers intended for incineration after use at the site. All study treatments are to be accounted for on the study treatment accountability log provided by the Sponsor. Unused supplies, as well as empty boxes of used supplies, will be either destroyed at the site or at the Interim Storage Facility. The Investigator should ensure adequate records are maintained via the study treatment accountability log.

## 9.6 Concomitant Medication/Therapy

The following concomitant medications are not permitted during this study (see also Section 6.2):

- Botulinum toxin other than that administered during the study.
- Any investigational new drug or device.
- Aminoglycoside antibiotics, curare-like non-depolarising blockers or other drugs that interfere with neuromuscular transmission.

Concomitant medications for other purposes are allowed at the Investigator's discretion. It is recommended that the dosage for these medications is kept constant throughout the study. Where medically appropriate, all concomitant medications being taken by a subject at entry into the study should continue at the same dose.

If any oral medication given for CD is initiated prior to study entry, the regimen should be continued at the same frequency and dose until at least the Week 4 visit and, whenever possible, until the end of the study.

## 9.7 Treatment of Overdose of IMP

Excessive doses of BTX-A may produce distant and profound neuromuscular paralysis. Overdose may lead to an increased risk of the neurotoxin entering the bloodstream and may cause complications associated with the effects of oral botulinum poisoning (e.g. dysphagia and dysphonia). Respiratory support may be required where excessive doses cause paralysis of the respiratory muscles. There is

no specific antidote (antitoxin should not be expected to be beneficial) so general supportive care is advised. In the event of overdose, the subject should be medically monitored for any signs and/or symptoms of excessive muscle weakness and/or muscle paralysis. Symptomatic treatment should be instigated if necessary. The signs and/or symptoms of overdose may not present immediately following injection. Should accidental injection or oral ingestion occur, the person should be medically supervised for several weeks for any signs and symptoms of excessive muscle weakness or muscle paralysis.

The administration of the study treatment will be carried out by a physician trained and experienced in the treatment of subjects with BTX-A and in an appropriate clinical settings. Therefore, facilities and staff for resuscitation and the treatment of overdose of study treatment or other medical emergencies will be available.

Any appropriate treatment of overdose of study treatment will be determined by the Investigator according to the characteristics of the events and will be recorded in the subject's eCRF. An event resulting from an overdose of the study treatment is not considered as serious unless it meets the definition of a SAE and consequently should be reported on the SAE form (see Section 10.4).

# 10 ADVERSE EVENT REPORTING

An AE is the development of an undesirable medical condition or the deterioration of a pre-existing medical condition following or during exposure to a pharmaceutical product, whether or not considered causally related to the product. An undesirable medical condition can be symptoms (e.g. nausea, chest pain), signs (e.g. tachycardia, enlarged liver) or the abnormal results of an investigation (e.g. laboratory findings, electrocardiogram). In clinical studies an AE can include an undesirable medical condition occurring at any time, including run-in or washout periods, even if no study treatment has been administered.

This definition includes events occurring from the time of the subject giving informed consent until the end of the study (as defined in Section 4.3.2).

## 10.1 Disease Progression

There is no disease progression associated with this indication.

## 10.2 Categorisation of Adverse Events

### *10.2.1 Intensity Classification*

AEs will be classified as mild, moderate or severe according to the following criteria:

| | |
|---|---|
| Mild: | symptoms do not alter the subject's normal functioning |
| Moderate: | symptoms produce some degree of impairment to function, but are not hazardous, uncomfortable or embarrassing to the subject |
| Severe: | symptoms definitely hazardous to well-being, significant impairment of function or incapacitation. |

### *10.2.2 Causality Classification*

The relationship of an AE to the study treatment will be classified according to the following:

Related: reports including good reasons and sufficient information (e.g. plausible time sequence, dose-response relationship, pharmacology, positive de-challenge and/or re-challenge) to assume a causal relationship with the study treatment in the sense that it is plausible, conceivable or likely.

Not related: reports including good reasons and sufficient information (e.g. implausible time sequence and/or attributable to concurrent disease or other drugs) to rule out a causal relationship with the study treatment.

#### *10.2.3 Assessment of Expectedness*

The expectedness of an AE/reaction shall be determined by the Sponsor according to the Investigator's Brochure for an unapproved study treatment or Package Insert (PI) for an authorised medicinal product which is being used according to the terms and conditions of the marketing authorisation. If the study treatment has marketing authorisations in several countries with different PIs, one will be selected as the reference document for assessing expectedness.

The reference document for assessing expectedness of AEs/reactions in this study will be the Dysport Therapeutic Indications Investigator's Brochure in effect at the time of the event [17].

#### *10.2.4 Laboratory Test Abnormalities*

Abnormalities in laboratory test values should only be reported as AEs if any of the following apply:

- They result in a change in study treatment schedule of administration (change in dosage, delay in administration, study treatment discontinuation).
- They require intervention or a diagnosis evaluation to assess the risk to the subject.
- They are considered as clinically significant by the Investigator.

#### *10.2.5 Abnormal Physical Examination Findings*

Clinically significant changes, in the judgement of the Investigator, in physical examination findings (abnormalities) will be recorded as AEs.

#### *10.2.6 Suicidality Assessment*

If, after completing the C-SSRS, a subject is determined to have experienced suicidal ideation, suicidal behaviour or related events, the event should be captured as an AE or SAE, in accordance with conditions of the Protocol. Events that meet serious criteria should be handled as described in Section 10.4.

**Note:** If, upon completing the C-SSRS, a subject is determined to have active suicidal thoughts and is in danger of personal harm, the principal Investigator should immediately take the appropriate steps to ensure subject safety, which may include referral and transfer to the appropriate psychiatric facility or department, or admission to an in-patient facility where subjects can be monitored and treated appropriately.

#### *10.2.7 Other Investigation Abnormal Findings*

Abnormal objective test findings as judged by the Investigator as clinically significant (e.g. electrocardiogram changes, laboratory test abnormalities) that result in a change in study treatment dosage or administration schedule, or in discontinuation of the study treatment, or require intervention or diagnostic evaluation to assess the risk to the subject, should be recorded as AEs

### 10.3 Recording and Follow Up of Adverse Events

At each visit the subject should be asked a non-leading question such as: "Do you feel different in any way since starting the new treatment/the last assessment?"

All observed or volunteered AEs, regardless of treatment group or suspected causal relationship to study treatment, will be recorded on the AE page(s) of the eCRF. Events involving drug reactions, accidents, illnesses with onset during the study, or exacerbations of pre-existing illnesses should be recorded.

Adverse events already recorded and designated as 'continuing' should be reviewed at each subsequent assessment.

For all AEs, the Investigator must pursue and obtain information adequate both to determine the outcome of the AE and to assess whether it meets the criteria for classification as a SAE requiring immediate notification to the Sponsor or its designated representative. For all AEs, sufficient information should be obtained by the Investigator to determine the causality of the AE (i.e. study treatment or other illness). The Investigator is required to assess causality and record that assessment on the eCRF. Follow up of the AE, after the date of therapy discontinuation, is required if the AE or its sequelae persist. Follow up is required until the event or its sequelae resolve or stabilise at a level acceptable to the Investigator and the Sponsor's clinical monitor or his/her designated representative.

Any SAE with a suspected causal relationship to the study treatment occurring at any other time after completion of the study must be promptly reported.

### 10.4 Serious Adverse Events

#### *10.4.1 Definitions*

All SAEs (as defined below) regardless of treatment group or suspected relationship to study treatment must be reported immediately (within 24 hours of the Investigator's knowledge of the event) to the Sponsor's Pharmacovigilance Department. If the immediate report is submitted by telephone, this must be followed by detailed written reports using the SAE report form.

A SAE is any AE occurring at any dose that:

(1) Results in death.

(2) Is life threatening, that is any event that places the subject at immediate risk of death from the reaction as it occurred. It does not include a reaction that, had it occurred in a more severe form, might have caused death.

(3) Results in in-patient hospitalisation or prolongation of existing hospitalisation, excluding admission for social or administrative reasons (see further).

(4) Results in a persistent or significant disability/incapacity, where disability is a substantial disruption of a person's ability to conduct normal life functions.

(5) Results in congenital anomaly/birth defect in the offspring of a subject who received the study treatment.

(6) Is an important medical event that may not result in death, be life-threatening, or require hospitalisation when, based upon appropriate medical judgement, may jeopardise the subject and may require medical or surgical intervention to prevent one of the outcomes listed in this definition. Examples of such medical events include allergic bronchospasm requiring intensive treatment in an emergency room or at home, blood dyscrasias or convulsions that do not result in in-patient hospitalisation, or the development of drug dependency or drug abuse.

Regardless of the above criteria, any additional AE that the Sponsor or an Investigator considers serious should be immediately reported to the Sponsor and included in the corporate SAEs database system.

- **Hospitalisation** is defined as any in-patient admission (even if less than 24 hours). For chronic or long-term in-patients, in-patient admission also includes transfer within the hospital to an acute/intensive care in-patient unit.
- **Prolongation of hospitalisation** is defined as any extension of an in-patient hospitalisation beyond the stay anticipated/required in relation to the original reason for the initial admission, **as determined by the Investigator or treating physician**. For protocol-specified hospitalisation in clinical studies, prolongation is defined as any extension beyond the length of stay described in the protocol. Prolongation in the absence of a precipitating, treatment emergent, clinical AE (i.e. not associated with the development of a new AE or worsening of a pre-existing condition) may meet criteria for "seriousness" but is not an adverse experience and thus is not subject to immediate reporting to the Sponsor.
- Pre-planned or elective treatments/surgical procedures should be noted in the subject's screening documentation. Hospitalisation for a pre-planned or elective treatment/surgical procedure should not be reported as an SAE unless there are complications or sequelae which meet the criteria for seriousness described above.

### *10.4.2 Reporting Requirements*

Any SAE must be reported immediately (within 24 hours), independent of the circumstances or suspected cause, if it occurs or comes to the attention of the Investigator at any time during the study period.

Any SAE with a suspected causal relationship to the study treatment occurring at any other time after completion of the study must be promptly reported.

### *10.4.3 Mandatory Information for Reporting an SAE*

The following information is the minimum that must be provided to the Sponsor pharmacovigilance contact within 24 hours for each SAE:

- Study number.
- Centre number.
- Subject number.
- Adverse event.
- Investigator's name and contact details.

The additional information included in the SAE form must be provided to the Sponsor or representative as soon as it is available. Upon receipt of the initial report, the Sponsor will ask for the Investigator's causality assessment if it was not provided with the initial report.

The Investigator should report a diagnosis or a syndrome rather than individual signs or symptoms. The Investigator should also try to separate a primary AE considered as the foremost untoward medical occurrence from secondary AEs which occurred as complications.

#### *10.4.4 Reporting Exemptions*

Tertiary efficacy endpoints will be measured using pain assessment scales (i.e. NRS and the BPI) and a depression scale (i.e. PHQ-9). As pain and depression are identified as tertiary endpoints for the indication under study, they will not be collected as AEs unless the pain or depression worsens and it is determined that the worsening was caused by the study drug.

### 10.5 Pregnancy

Pregnancy itself is not regarded as an AE unless there is a suspicion that the study treatment has interfered with a contraceptive method. If pregnancy occurs following exposure to study treatment, then the subject must be immediately withdrawn from the treatment phase of the study. The outcome of the pregnancy will then need to be collected even if this occurs after the end of the study.

Information regarding pregnancies must be collected on the AE page of the eCRF and reported to the sponsor as an SAE. The Sponsor will request further information from the Investigator as to the course and outcome of the pregnancy using the Standard Pregnancy Outcome Report Form.

Investigators must instruct all female subjects to inform them immediately should they become pregnant during the study. The Investigator should counsel the subject, discuss the risks of continuing with the pregnancy and the possible effects on the foetus. Monitoring of the subject should continue until conclusion of the pregnancy, which may involve follow up after the subject’s involvement in the study has ended.

In the event of early withdrawal, pregnancies with a conception date within 90 days of dosing the subject with study treatment must also be reported to the Investigator for onward reporting to the Sponsor.

### 10.6 Deaths

All AEs resulting in death either during the study period or within 3 months after the last dose of study treatment, must be reported as an SAE within 24 hours of the Investigator’s knowledge of the event.

The convention for recording death is as follows:

- Adverse event term: lead cause of death (e.g. multiple organ failure, pneumonia, myocardial infarction).
- Outcome: fatal.
- The **only exception** is if the cause of death is unknown (i.e. sudden or unexplained death), in which case the AE term may be ‘Death’ or ‘Sudden death’.

## 10.7 Discontinuation/Withdrawal due to Adverse Events/Serious Adverse Events

Discontinuation/withdrawal due to AEs should be distinguished from discontinuation/withdrawal due to insufficient response to the study treatment (see Sections 6.4 and 6.5).

If the study treatment is discontinued due to a SAE it must be reported immediately to the Sponsor's designated representative (see Section 10.4).

In all cases the Investigator must ensure the subject receives appropriate medical follow up (see Section 10.3).

## 10.8 Reporting to IRBs/Other Investigators

The Sponsor will ensure that processes are in place for submission of reports of Suspected Unexpected Serious Adverse Reactions occurring during the study to the IRBs and other Investigators concerned by the study treatment. Reporting will be done in accordance with the applicable regulatory requirements.

For study centres in the USA, Investigational New Drug (IND) Safety Reports will be submitted directly to the Investigators. It is the Investigators' responsibility to notify their IRB in a timely manner.

# 11 STATISTICAL CONSIDERATIONS

## 11.1 Subject Classification and Definitions

| | |
|---|---|
| **Enrolled subject:** | Subject fully informed about the study who has given written informed consent to participate (before any occurrence of study related procedure). |
| **Screened failure subject:** | Enrolled subject who fails to fulfil one or more entry criteria and thus does not proceed to the treatment phase of the study. Although not exposed to study treatment, they may have been exposed to some study related procedures. Records up to the time of premature termination should be completed including the reason for termination. |
| **Treated subject:** | Enrolled subject who is treated with at least one dose of study treatment. |
| **Randomised subject:** | Enrolled subject who is allocated to a treatment group at random. |
| **Treatment completed subject:** | Treated subject who has completed all specified assessments of the treatment. |
| **Study completed subject:** | Treated subject who has completed all specified assessments of the study. |
| **Drop-out:** | Randomised subject who did not complete the study. |

## 11.2 Analyses Populations Definitions

| | |
|---|---|
| **Screened population:** | All subjects enrolled. |
| **Randomised population:** | All subjects randomised. |
| **Safety population:** | All randomised subjects who received study treatment regardless of the amount of study treatment administered and had at least one follow up safety assessment. |
| **Intent to treat (ITT) population:** | All subjects randomised. |
| **Modified intent to treat population (mITT):** | A subset of the ITT population, composed of randomised subjects with both a baseline and a Week 4 post-treatment TWSTRS total score assessment. |
| **Per protocol (PP) population:** | All subjects in the mITT population who were not major protocol violators. |

### *11.2.1 Populations Analysed*

The primary efficacy analysis will be performed on the mITT population and confirmed using the PP population .
The analyses of safety data will be performed based on the safety population.

### *11.2.2 Subject Allocation and Reasons for Exclusion from the Analyses*

The rules for the allocation of subjects to each of the analysis populations will be defined in the Reporting and Analysis Plan (RAP) and finalised during a "blind" review meeting held prior to database lock, before any unblinding of treatment groups.
During the blind/data review meeting, based on minor or major protocol violations/deviations, subjects may be excluded from the analysis populations of interest.
Subjects may be excluded from the analyses if one or more of the following violations/deviations occur:

- Randomisation criteria violations.
- Inclusion/exclusion criteria violations.
- Did not receive any study treatment.
- In adequate compliance with study treatment.
- Prohibited medication intake.
- Deviations from time windows.
- Deviations from study treatment administration.
- No baseline evaluation of primary efficacy criterion.
- No valid post baseline evaluation of primary efficacy criterion.
- Other protocol violation/deviations.

## 11.3 Sample Size Determination

The number of 132 randomised subjects (i.e. 88 subjects in the Dysport group and 44 subjects in the placebo group) is considered sufficient to demonstrate the superiority of Dysport to placebo assuming a minimum clinically relevant difference in the adjusted least squares mean change from baseline in TWSTRS total score at

Week 4 between Dysport 2 mL and placebo equal to 5.5, a common standard deviation in the change from baseline in TWSTRS total score at Week 4 equal to 8.8, a power of 90%, a two-tailed type I error equal to 0.05 and 10% drop out rate.

#### *11.3.1 Significance Testing and Estimations*

All statistical tests will be performed two sided with a type I error rate set at 5%.

### 11.4 Statistical/Analytical Methods

Statistical analyses will be performed by an external Contract Research Organisation (CRO), managed by the Sponsor's Statistics Department.
A RAP describing the planned statistical analysis in detail with tables, figures and listings templates will be developed as a separate document.
Statistical evaluation will be performed using Statistical Analysis System (SAS)® (version 9 or higher).

#### *11.4.1 Demographic and Other Baseline Characteristics*

All demographic and baseline characteristics will be listed by treatment group and subject identification. Summary statistics for demographic and baseline characteristics will be presented by treatment group and overall for the ITT, mITT and PP populations.

##### *11.4.1.1 Homogeneity of Treatment Groups*

The comparability between treatment groups for demographic and baseline characteristics may be assessed using inferential statistical testing.

##### *11.4.1.2 Subject Disposition and Withdrawals*

The distribution of subjects enrolled and included in each of the ITT, mITT and PP populations will be tabulated by centre. The reasons for subject exclusions from each of the populations will also be tabulated. In addition, the numbers of subjects who were randomised/treated, discontinued and completed will be summarised by treatment group. Primary reasons for discontinuation of study treatment will be tabulated.

#### *11.4.2 Pharmacokinetic Data*

There are no pharmacokinetic analyses planned for this study.

#### *11.4.3 Efficacy Evaluation*

As indicated in Section 8.1.1, the primary efficacy endpoint is the change from baseline in TWSTRS total score at Week 4. Secondary efficacy endpoints (see Section 8.1.2) are ranked in hierarchical order to test for the superiority of Dysport to placebo in a robust manner. The ranks of the secondary endpoints are:

(1) Change from baseline in TWSTRS total score at the Week 2 visit.

(2) Clinical Global Impression of Change assessment of CD change at the Week 2 visit.

(3) Treatment response at the Week 2 visit. A treatment responder is defined as a subject who had at least a 30% reduction in the TWSTRS total score after treatment.

(4) Clinical Global Impression of Change assessment of CD change at the Week 4 visit.

(5) Treatment response at the Week 4 visit.
(6) Change from baseline in CDIP-58 score at the Week 4 visit.
(7) Change from baseline in CDIP-58 score at the Week 2 visit.

A hierarchical testing procedure combining the primary and ranked secondary efficacy endpoints together with type I error controlled at level of 5% ('study-wise' including both primary and secondary efficacy endpoints).

The hierarchical testing procedure will be performed as follows:

- Step 1: The superiority of Dysport to placebo on the primary efficacy endpoint will be tested at a two-tailed 5% level by using a stratified analysis of covariance (ANCOVA) with baseline TWSTRS total score as covariate and stratified by the randomisation stratification factor (BTX naïve versus BTX non-naïve). Specifically, the treatment differences, $D_n$ and $D_b$, will first be calculated from the ANCOVA model for naive subjects and subjects who were on prior BTX, respectively. Then the sample size weighted overall treatment difference, D, will be calculated as $D = w_nD_n + w_bD_b$ where $w_n = n_n/n$ and $w_b = n_b/n$ ($n_n$ is the number of naive subjects, $n_b$ is the number of subjects who are on prior therapy with BTX, and $n = n_n + n_b$). The hierarchical testing procedure will be stopped if there is no statistically significant treatment effect on the primary efficacy endpoint. Otherwise Step 2 will be performed.
- Step 2: The superiority of Dysport to placebo on the rank-1 secondary efficacy endpoint will be tested using the same method as the one used for the primary efficacy endpoint. The hierarchical testing procedure will be stopped if there is no statistically significant treatment effect on the current efficacy endpoint. Otherwise the next step will be performed.
- Step 3: The superiority of Dysport to placebo on CGIC of CD at the Week 2 visit will be tested at a two-tailed 5% level by using an analysis of variance (ANOVA) with treatment and randomisation stratification factor as main effect. The hierarchical testing procedure will be stopped if there is no statistically significant treatment effect on the current efficacy endpoint. Otherwise the next step will be performed.
- Step 4: The superiority of Dysport to placebo on treatment response at Week 2 will be tested at a two-tailed 5% level by using Mantel-Haenszel chi-squared test stratified by the randomisation stratification factor. The hierarchical testing procedure will be stopped if there is no statistically significant treatment effect on the current efficacy endpoint. Otherwise the next step will be performed.
- Step 5: The superiority of Dysport to placebo on CGIC of CD at the Week 4 visit will be tested at a two-tailed 5% level by using an ANOVA with treatment and randomisation stratification factor as main effect. The hierarchical testing procedure will be stopped if there is no statistically significant treatment effect on the current efficacy endpoint. Otherwise the next step will be performed.
- Step 6: The superiority of Dysport to placebo on treatment response at Week 4 will be tested at a two-tailed 5% level by using Mantel-Haenszel chi-squared test stratified by the randomisation stratification factor. The

hierarchical testing procedure will be stopped if there is no statistically significant treatment effect on the current efficacy endpoint. Otherwise the next step will be performed.

- Step 7: The superiority of Dysport to placebo on CDIP-58 at the Week 4 visit will be tested at a two-tailed 5% level by using an ANOVA with treatment and randomisation stratification factor as main effect. The hierarchical testing procedure will be stopped if there is no statistically significant treatment effect on the current efficacy endpoint. Otherwise the next step will be performed.
- Step 8: The superiority of Dysport to placebo on CDIP-58 at the Week 2 visit will be tested at a two-tailed 5% level by using an ANOVA with treatment and the randomisation stratification factor as the main effects.

If the hierarchical testing procedure is prematurely stopped (prior to Step 8) then the analysis of remaining secondary efficacy endpoint(s) will be performed to characterise the full clinical effect but no formal statistical conclusion will be drawn.

Exploratory analysis will be performed for each of the tertiary secondary efficacy endpoints using appropriate methods. The tertiary secondary efficacy endpoints are:

- Change from baseline in TWSTRS severity, disability and pain subscale scores at the Week 2, 4 and 12 visits.
- Change from baseline in TWSTRS total score at the Week 12 visit.
- Patient Global Impression of Change assessment of CD change at the Week 2, 4 and 12 visits.
- Change from baseline in pain NRS at the Week 2, 4 and 12 visits.
- Change from baseline in BPI short form at the Week 2, 4 and 12 visits.
- Change from baseline in PHQ-9 at the Week 2, 4 and 12 visits.
- Modified TSQM-9 change at the Week 2, 4 and 12 visits.
- Clinical Global Impression of Change assessment of CD change at the Week 12 visit.
- Treatment response at the Week 12 visit.
- Change from baseline in CDIP-58 score at the Week 12 visit.

#### *11.4.4 Adjustment for Country/Centre Effect*

Due to the small number of subjects enrolled in each centre, the analysis will be pooled. However, the treatment by centre interaction will be investigated by plotting the treatment differences (i.e. least square mean differences) by centre to check the homogeneity of treatment responses between centres. A formal ANCOVA model, with treatment by centre, will also be evaluated with a nominal 20% significance level.

#### *11.4.5 Safety Evaluation*

All safety data will be included in the subject data listings. Analyses and summary tables will be based upon the safety population.

***Adverse Events***

Adverse events will be coded according to the Medical Dictionary for Regulatory Activities (MedDRA) version in force within Ipsen Coding department at the time of the database freeze and will be classified by MedDRA preferred term and system

organ class. Adverse event listings will be presented by subject, system organ class and preferred term.

Incidence of all reported AEs/TEAEs and SAEs will be tabulated by treatment group. In addition, summary tables will be presented by maximum intensity, drug relationship and TEAEs associated with premature withdrawal of study treatment.

A TEAE is defined as any AE that occurs during the active phase of the study if:

(1) it was not present prior to receiving the first dose of study treatment, or
(2) it was present prior to receiving the first dose of study treatment but the intensity increased during the active phase of the study, or
(3) it was present prior to receiving the first dose of study treatment, the intensity is the same but the drug relationship became related during the active phase of the study.

Treatment emergent AEs will be flagged (*) in the AEs listings.

***Suicidality Data***

The differences in the proportion of subjects reporting any suicidality (behaviour and ideation) between the Dysport and the placebo groups will be summarised and compared using Fisher's Exact test [24].

***Prior and Concomitant Medications***

Prior and concomitant medication will be coded by using World Health Organisation Drug Dictionary (WHO-Drug), version in force within Ipsen Coding department at the time of the database freeze, and will be summarised by treatment group with the number and percentage of subjects receiving concomitant medication by drug class and preferred drug name.

***Vital Signs***

Summary statistics (mean, median, standard deviation and range) by treatment group will be presented for sitting BP, HR, oral temperature and respiratory rate at each assessment with change from baseline.

***Electrocardiograms***

No electrocardiograms will be performed in this study.

***Laboratory Parameters***

For clinical laboratory safety tests (biochemistry), all the abnormal values will be flagged in the data listings and a list of clinically significant abnormal values will be presented by treatment group and subject ID for evaluations at baseline and end of study or early withdrawal from the study.

Shift from baseline tables of the number and percentage of subjects with low, normal or high values will also be presented for each treatment group.

***Pregnancy Test***

A urine sample will be collected for a pregnancy test at screening for female subjects of childbearing potential. If this is found to be positive, the subject will be excluded from the study. A urine sample will also be collected for a pregnancy test at the end of study/early withdrawal visit. The pregnancy test results will be listed by treatment group and subject identification.

### 11.5 Subgroup Analyses

Subgroup analysis according to the randomisation stratification factor (BTX naïve subjects versus BTX non-naïve subjects) may be considered in this study.

### 11.6 Interim Analyses and Data Monitoring

No interim analysis will be performed.

### 11.7 Final Analysis

The final analysis will be performed after all the subjects finish the study.

## 12 MONITORING PROCEDURES

The Investigator is responsible for the validity of all data collected at the site. The Sponsor is responsible for monitoring this data to verify that the rights and well being of subjects are protected, that study data are accurate (complete and verifiable to source data) and that the study is conducted in compliance with the protocol, GCP and regulatory requirements.

### 12.1 Routine Monitoring

Sponsor-assigned monitors will conduct regular site visits. The Investigator will allow direct access to all relevant files (for all subjects) and clinical study supplies (dispensing and storage areas) for the purpose of verifying entries made in the eCRF, and assist with the monitor's activities, if requested. Adequate time and space for monitoring visits should be made available by the Investigator.

The site must complete the eCRFs within 5 days of the subject's visit and on an ongoing basis to allow regular review by the study monitor, both remotely via the internet and during site visits. The central study monitor at IPSEN will use functions of the EDC system to address any queries raised while reviewing the data entered by the study site personnel in a timely manner.

Whenever a subject name is revealed on a document required by the Sponsor (e.g. laboratory print-outs) the name must be blacked out permanently by the site personnel, leaving the initials visible, and annotated with the subject number as identification.

## 13 STUDY MANAGEMENT

### 13.1 Inspections and Auditing Procedures

Authorised personnel from Sponsor-authorised Quality Assurance personnel may carry out inspections and audits. The purpose of an audit is to ensure that ethical, regulatory and quality requirements are fulfilled in all studies performed by the Sponsor.

Auditors and inspectors must have direct access to study documents and site facilities as specified in section 12.1, and to any other locations used for the purpose of the study in question (e.g. laboratories).

In the event of the site being notified directly of a regulatory inspection, the Investigator must notify the Sponsor representative as soon as possible, to assist with preparations for the inspection.

### 13.2 Data Recording of Study Data

In compliance with GCP, the medical records/medical notes, etc., should be clearly marked and permit easy identification of a subject's participation in the specified clinical study.

The Investigator must record all data relating to protocol procedures, study treatment administration, safety data and efficacy ratings on the eCRFs provided for the study. The Investigator, by completing the signature log, may formally designate authority to complete eCRFs to appropriately qualified staff having certified user access to the eCRF. Subject completed diaries and questionnaires will be printed or electronic.

The Investigator must, as a minimum, provide an electronic signature (e-signature) to each "visit status" eCRF page to attest to the accuracy and completeness of all the data. If any changes are made to the eCRF, after a form has been locked and electronically signed, the Investigator will be required to perform an additional e-signature authorising agreement with any new information or changes to the eCRF.

All corrections on the eCRF will be automatically tracked and a reason for change is always required. In the eCRF, the audit trail function will allow the changes made to be viewed on each item entered.

## 13.3 Source Data Verification

The FDA 21 CFR Part 11, is a regulation which provides criteria for acceptance by the FDA, under certain circumstances, of electronic records, e-signatures and hand-written signatures executed to electronic records as equivalent to paper records and hand-written signatures on paper.

As required by GCP, the Sponsor assigned monitor must verify, by direct reference to the source documents, that the data required by the protocol are accurately reported on the eCRF.

As required by ICH GCP §6.4.9, if some items are recorded directly on the eCRF and are considered as source data, the identification of these data must be documented and agreed between the Investigator and the Sponsor.

The source documents must, as a minimum, contain the following; a statement that the subject is included in a clinical study, the date that informed consent was obtained prior to participation in the study, the identity of the study, diagnosis and eligibility criteria, visit dates (with subject status), study treatment administration, and any AEs and associated concomitant medication.

Definition for source data and source documents are given below:

**Source Data:** All original records and certified copies of original records of clinical findings, observations, or other activities necessary for the reconstruction and evaluation of the study. Source data are contained in source documents (original records or certified copies). (ICH GCP Section 1.51).

**Source Documents:** Original documents, data and records (e.g. hospital records, clinical and office charts, laboratory notes, memoranda, subjects' diaries or evaluation checklists, pharmacy dispensing records, recorded data from automated instruments, copies or transcriptions certified after verification as being accurate copies, microfiches, photographic negatives, microfilm or magnetic media, X-rays, subject files, and records kept at the pharmacy, at the laboratories and at medico-technical departments involved in the clinical study). (ICH GCP Section 1.52).

The subject must have consented to their medical records being viewed by Sponsor-authorised personnel. This information is included in the informed consent.

## 13.4 Data Quality

Monitored eCRFs transferred from the investigational site to the assigned Data Management group will be reviewed (secondary monitoring) for completeness, consistency, legibility and protocol compliance.

Reasons should be given on the relevant eCRF for any missing data and other protocol deviations. Any electronic queries and items not adequately explained will require additional electronic manual queries to be raised to the Investigator by the monitor for clarification/correction. The Investigator must ensure that queries are dealt with promptly. All data changes and clarifications can be viewed in the audit trail function of the eCRF.

## 13.5 Data Management

Electronic Data Capture (EDC) will be utilised for collecting subject data. Each site is required to have a computer and internet connection available for site entry of clinical data. All entries in the eCRF will be done under the electronic signature of the person performing the action. This electronic signature consists of an individual and confidential username and password combination. It is declared to be the legally binding equivalent of the handwritten signature. Only Sponsor authorised users will get access to the eCRF as appropriate to their study responsibilities. Users must have successfully undergone software application training prior to entering data into the eCRF.

Paper CRFs will be available to ensure business continuity in case the eCRFs are unavailable at the site for a prolonged period, they will be used only after prior permission is gained from the Sponsor.

Data management will be conducted by a CRO, directed by the Sponsor's Statistics Department. All data management procedures will be completed in accordance with Ipsen and the contracted CRO SOPs. Prior to data being received in-house at the assigned CRO, it will be monitored at the Investigator site, (for further details please see Section 12 Monitoring Procedures). eCRF and other data documentation removed from the Investigator site(s) will be tracked by the CRO and the monitor.

The Sponsor will ensure that an appropriate eCRF is developed to capture the data accurately, and suitable queries are raised to resolve any missing or inconsistent data. The Investigator will receive their data, from the clinical study, in an electronic

format (PDF files) which will be an exact copy of the eCRF, and will include the full audit trail, for archiving purposes and future reference.

Any queries generated during the data management process will also be tracked by the contracted data management CRO/will be raised within the EDC system. It is the central study monitor's responsibility to ensure that all queries are resolved by the relevant parties.

The Sponsor will also ensure that SAE data collected in the eCRF are consistent with information provided to the Sponsor's pharmacovigilance department (and vice versa).

The coding of an AE, medical history and concomitant medication terms will be performed by the contracted CRO, directed by the Sponsor's Statistics Department, and reviewed and approved by the Sponsor. Concomitant medications will be coded using WHO-Drug and AEs/medical history terms will be coded using MedDRA.

## 13.6 Study Management Committees

### *13.6.1 Steering Committee*

The Steering Committee will be composed of Investigator and Sponsor representatives to govern the overall scientific and operational management of the study. A specific charter may be developed to define roles and responsibilities.

## 13.7 Record Archiving and Retention

During the pre-study and initiation visits, the monitor must ensure the archiving facilities are adequate and archiving/retention responsibilities of the Investigator have been discussed.

Study documents should be retained until at least 2 years after the last approval of a marketing application in an ICH region and until there are no pending or planned marketing applications in an ICH region (that is at least 15 years) or at least 2 years have elapsed since the formal discontinuation of clinical development of the product. However, these documents should be retained for a longer period if required by the applicable regulatory requirements or by an agreement with the Sponsor. The Investigator should take measures to prevent accidental or premature destruction of these documents. The final archiving arrangements will be confirmed by the monitor when closing-out the site. The Sponsor will inform the Investigator, in writing, as to when these documents no longer need to be retained.

If the Principal Investigator relocates or retires, or otherwise withdraws his/her responsibility for maintenance and retention of study documents, the Sponsor must be notified (preferably in writing) so that adequate provision can be made for their future maintenance and retention.

# 14 ADMINISTRATION PROCEDURES

## 14.1 Regulatory Approval

As required by local regulations, the Sponsor's Regulatory Affairs group will ensure all legal regulatory aspects are covered, and obtain approval of the appropriate regulatory bodies, prior to study initiation in regions where an approval is required.

## 14.2 Publication Policy

The Sponsor encourages acknowledgement of all individuals/organisations involved in the funding or conduct of the study, including medical writers or statisticians subject to the consent of each individual and entity concerned, including acknowledgement of the Sponsor.

The results of this study may be published or communicated to scientific meetings by the Investigators involved in the study. For multicentre studies, a plan for scientific publication and presentation of the results may be agreed and implemented by the study Investigators or a Steering Committee. The Sponsor requires that reasonable opportunity be given to review the content and conclusions of any abstract, presentation, or paper before the material is submitted for publication or communicated. This condition also applies to any amendments that are subsequently requested by referees or journal editors. The Sponsor will undertake to comment on the draft documents within the time period agreed in the contractual arrangements, including clinical study agreements, governing the relationship between the Sponsor and authors (or the author's institution). Requested amendments will be incorporated by the author, provided they do not alter the scientific value of the material.

If patentability would be adversely affected by publication, this will be delayed until (i) a patent application is filed for the content of the publication in accordance with applicable provisions of the clinical study agreement concerned, (ii) the Sponsor consents to the publication, or (iii) the time period as may be agreed in the contractual arrangements, including clinical study agreements, governing the relationship between the Sponsor and authors (or authors' institution) after receipt of the proposed publication by the Sponsor, whichever of (i), (ii) or (iii) occurs first.

The author undertakes to reasonably consider the Sponsor's request for delay to the proposed publication should the Sponsor reasonably deem premature to publish the results obtained at the then stage of the study.

## 14.3 Clinical Study Report

A final clinical study report will be prepared according to the ICH guideline on structure and contents of clinical study reports. A final clinical study report will be prepared where any subject has signed informed consent, regardless of whether the study is completed or prematurely terminated. Where appropriate an abbreviated report may be prepared. The CSR will be in compliance with any applicable regulatory requirements, national laws in force and will be in English.

## 14.4 Contractual and Financial Details

The Investigator (and/or, as appropriate, the hospital administrative representative) and the Sponsor will sign a clinical study agreement prior to the start of the study, outlining overall Sponsor and Investigator responsibilities in relation to the study. Financial remuneration will cover the cost per included subject, based on the calculated costs of performing the study assessments in accordance with the protocol, and the specified terms of payment will be described in the contract. The contract should describe whether costs for pharmacy, laboratory and other protocol-required services are being paid directly or indirectly.

Financial Disclosure Statements will need to be completed, as requested by FDA 21 CFR Part 54.

### 14.5 Insurance, Indemnity and Compensation

The Sponsor will provide Product Liability insurance for all subjects included in the clinical study. Where required, a hospital specific indemnity agreement will be used.

## 15 PROTOCOL AMENDMENTS

Protocol amendments shall be submitted to the relevant IND and IRB(s).

# 16 REFERENCES


1 Nutt JG, Muenter MD, Aronson A, Kurland LT, Melton LJ 3rd. Epidemiology of focal and generalized dystonia in Rochester, Minnesota. Mov Disord 1988;3(3):188-194.

2 Chan J, Brin MF, Fahn S. Idiopathic cervical dystonia, clinical characteristics. Mov Disord 1991;6(2):119-126.

3 Jankovic J, Leder S, Warner D, Schwartz K. Cervical dystonia: clinical findings and associated movement disorders. Neurology 1991;41(7):1088-1091.

4 Rondot P, Marchand MP, Dellatolas G. Spasmodic torticollis, review of 220 patients. Can J Neurol Sci 1991;18(2):143-151.

5 Deuschl G, Heinen F, Kleedorfer B, Wagner M, Lucking CH, Poewe W. Clinical and polymyographic investigation of spasmodic torticollis. J Neurol 1992;239(1):9-15.

6 Consky ES, Lang AE. Clinical assessments of patients with cervical dystonia. In: Jankovic J, Hallet M, eds. Therapy with botulinum toxin. New York: Marcel Dekker Inc., 1994:211-237.

7 Jankovic J, Brin MF. Therapeutic uses of botulinum toxin. N Engl J Med 1991;324(17):1186-1194.

8 Coffield JA, Considine RV, Simpson LL. The site and mechanism of action of botulinum neurotoxin. In: Jankovic J, Hallett M, eds. Therapy with botulinum toxin. New York: Marcel Dekker Inc., 1994:3-14.

9 Odergren T, Tollbäck A, Borg J. Electromyographic single motor unit potentials after repeated botulinum toxin treatments in cervical dystonia. Electroencephalogr Clin Neurophysiol 1994;93(5):325-329.

10 Clinical Study Report, Study Y-97-52120-045.

11 Truong D, Duane DD, Jankovic J, et al. Efficacy and safety of botulinum type A toxin (Dysport) in cervical dystonia: results of the first US randomized, double-blind, placebo-controlled study. Mov Disord. 2005;20(7):783-791.

12 Clinical Study Report, Study Y-47-52120-051.

13 Truong D, Brodsky M, Lew M, et al. Long-term efficacy and safety of botulinum toxin type A (Dysport) in cervical dystonia. Parkinsonism Relat Disord 2010;16(5):316-323.

14 Poewe W, Deuschl G, Nebe A et al. What is the optimal dose of botulinum toxin A in the treatment of cervical dystonia? Results of a double blind, placebo controlled, dose ranging study using Dysport. German Dystonia Study Group. J Neurol Neurosurg Psychiatry 1998;64(1):13-17.

15 Clinical Study Report, Study Y-97-52120-042.

16 Wissel J, Kanovsky P, Ruzicka E et al. Efficacy and safety of a standardised 500 unit dose of Dysport® (Clostridium botulinum toxin type A haemaglutinin complex) in a heterogeneous cervical dystonia population: results of a prospective, multicentre, randomised, double-blind, placebo-controlled, parallel group study. J Neurol 2001:248:1073-1078.

17 Dysport Therapeutic Indications Investigator's Brochure.

18 Hauser R, Coleman C, Yair J, Beffy JL, Picaut P. AbobotulinumtoxinA (Dysport) Dosing in Cervical Dystonia: An Analysis of Two Large Open-Label Extension Studies. Presented at The 15th International Congress of Parkinson's Disease and Movement Disorders, Toronto, Ontario, Canada, 5–9 June 2011.

19 International Conference on Harmonisation (ICH) E6 and Federal register Vol 63, No. 179 (September 1998).

20 Food and Drug Administration (FDA) 21 CFR Part 11, Electronic Records, Electronic Signatures.

21 FDA, Guidance for Industry: Computerized Systems Used in Clinical Trials.

22 Cano SJ, Warner TT, Linacre JM, et al. Capturing the true burden of dystonia on patients: the Cervical Dystonia Impact Profile (CDIP-58). Neurology 2004;63(9):1629-1633.

23 Posner K, Oquendo MA, Gould M, Stanley B, Davies M. Columbia Classification Algorithm of Suicidal Assessment (C-CASA): classification of suicidal events in the FDA's pediatric suicidal risk analysis of antidepressants. Am J Psychiatry, 2007;164(7): 1035-1043.

24 Columbia-Suicide Severity Rating Scale (C-SSRS). Available on the Columbia University Medical Center website.

# 17 LIST OF APPENDICES

**Appendix 1: Sample TWSTRS** ........ 58
**Appendix 2: Clinical Global Impression of Change** ........ 64
**Appendix 3: CDIP-58 Questionnaire** ........ 66
**Appendix 4: Patient Global Impression of Change** ........ 69
**Appendix 5: Numeric Rating Scale: Subject Assessment of Pain** ........ 71
**Appendix 6: Brief Pain Inventory Short Form** ........ 73
**Appendix 7: Patient Health Questionnaire (PHQ)-9** ........ 75
**Appendix 8: Modified TSQM-9** ........ 77
**Appendix 9: C-SSRS - Already Enrolled Subjects** ........ 80
**Appendix 10: C-SSRS - Baseline/Screening** ........ 86
**Appendix 11: C-SSRS – Since Last Visit** ........ 91

# Appendix 1: Sample TWSTRS

## Sample Toronto Western Spasmodic Torticollis Rating Scale (TWSTRS)[6]

## I. Torticollis Severity Scale (Sum of A-F, Maximum = 35)

### I-A. Maximal Excursion

*Rate maximum amplitude of excursion asking subject not to oppose the abnormal movement: examiner may use distracting or aggravating manoeuvres. When degree of deviation is between two scores, choose the higher of the two.*

**1. Rotation (Turn: Right or Left)**

0 = None (0°)
1 = Slight (<¼ range, 1° – 22°)
2 = Mild (¼ – ½ range, 23° – 45°)
3 = Moderate (½ – ¾ range, 46° – 67°)
4 = Severe (>¾ range, 68° – 90°)

**2. Laterocollis (Tilt: Right or Left, Exclude Shoulder Elevation)**

0 = None (0°)
1 = Mild (1° – 15°)
2 = Moderate (16° – 35°)
3 = Severe (>35°)

**3. Anterocollis or Retrocollis (a or b)**

a. Anterocollis
0 = None
1 = Mild downward deviation of chin
2 = Moderate downward deviation (approximates ½ possible range)
3 = Severe (chin approximates chest)

b. Retrocollis
0 = None
1 = Mild backward deviation of vertex with upward deviation of chin
2 = Moderate backward deviation (approximates ½ possible range)
3 = Severe (approximates full range)

**4. Lateral Shift (Right or Left)**

0 = Absent
1 = Present

**5. Sagittal Shift (Forward or Backward)**

0 = Absent
1 = Present

### I-B. Duration Factor (Weighted x 2)

*Provide an overall score estimated through the course of the standardised examination after estimating maximal excursion (exclusive of asking subject to allow head to deviate maximally). Weighted x 2 (see schematic representation of scoring duration below).*

0 = None
1 = Occasional deviation (<25% of the time, most often submaximal)
2 = Occasional deviation (<25% of the time, often maximal)
or
Intermittent deviation (25% – 50% of the time, most often submaximal)

3 = Intermittent deviation (25% – 50% of the time, often maximal)
or
Frequent deviation (>50% – 75% of the time, most often submaximal)
4 = Frequent deviation (>50% – 75% of the time, often maximal)
or
Constant deviation (>75% of the time, most often submaximal)
5 = Constant deviation (>75% of the time, often maximal)

**Schematic Representation of Duration Scoring***

| | | |
|---|---|---|
| None | | 0 |
| <25% of time (occasional) | submaximal | 1 |
| | maximal | 2 |
| 25 - 50% (intermittent) | submaximal | 2 |
| | maximal | 3 |
| >50 - 75% (frequent) | submaximal | 3 |
| | maximal | 4 |
| >75% (constant) | submaximal | 4 |
| | maximal | 5 |

*The rater simply determines the proportion of time that the dystonic head posturing is present (left column) and then decides whether the deviations are most often maximal or submaximal, having previously determined the maximal excursion score (IA).

**I-C. Effect of Sensory Tricks**

0 = Complete relief by one or more tricks
1 = Partial or only limited relief by tricks
2 = Little or no benefit from tricks

**I-D. Shoulder Elevation/Anterior Displacement**

0 = Absent
1 = Mild (<⅓ possible range, intermittent or constant)
2 = Moderate (⅓ – ⅔ possible range and constant, >75% of the time)
or
Severe (>⅔ possible range and intermittent)
3 = Severe and constant

**I-E. Range of Motion (Without Aid of Sensory Tricks)**

*If limitation occurs in more than one plane of motion use individual score that is highest.*

0 = Able to move to extreme opposite position
1 = Able to move head well past midline but not to extreme opposite position
2 = Able to move head barely past midline
3 = Able to move head toward but not past midline
4 = Barely able to move head beyond abnormal posture

**I-F. Time (Up to 60 Seconds) for Which Subject is Able to Maintain Head Within 10° of Neutral Position Without Using Sensory Tricks (Mean of Two Attempts)**

0 = >60 seconds
1 = 46 – 60 seconds
2 = 31 – 45 seconds
3 = 16 – 30 seconds
4 = <15 seconds

**II. Disability Scale (Sum of A-F, Maximum = 30)**

**II-A. Work (Occupation or Housework/Home Management)**

0 = No difficulty
1 = Normal work expectations with satisfactory performance at usual level of occupation but some interference by torticollis
2 = Most activities unlimited, selected activities very difficult and hampered but still possible with satisfactory performance
3 = Working at lower than usual occupation level; most activities hampered, all possible but with less than satisfactory performance in some activities
4 = Unable to engage in voluntary or gainful employment; still able to perform some domestic responsibilities satisfactorily
5 = Marginal or no ability to perform domestic responsibilities

**II-B. Activities of Daily Living (e.g. Feeding, Dressing, or Hygiene, Including Washing, Shaving, Makeup, etc.)**

0 = No difficulty with any activity
1 = Activities unlimited but some interference by torticollis
2 = Most activities unlimited, selected activities very difficult and hampered but still possible using simple tricks
3 = Most activities hampered or laborious but still possible; may use extreme tricks
4 = All activities impaired; some impossible or require assistance
5 = Dependent on others in most self-care tasks

**II-C. Driving**

0 = No difficulty (or has never driven a car)
1 = Unlimited ability to drive but bothered by torticollis
2 = Unlimited ability to drive but requires tricks (including touching or holding face, holding head against head rest) to control torticollis
3 = Can drive only short distances
4 = Usually cannot drive because of torticollis
5 = Unable to drive and cannot ride in a car for long stretches as a passenger because of torticollis

**II-D. Reading**

0 = No difficulty
1 = Unlimited ability to read in normal seated position but bothered by torticollis
2 = Unlimited ability to read in normal seated position but requires use of tricks to control torticollis
3 = Unlimited ability to read but requires extensive measures to control torticollis or is able to read only in nonseated position (e.g. lying down)
4 = Limited ability to read because of torticollis despite tricks
5 = Unable to read more than a few sentences because of torticollis

**II-E. Television**

0 = No difficulty
1 = Unlimited ability to watch television in normal seated position but bothered by torticollis
2 = Unlimited ability to watch television in normal seated position but requires use of tricks to control torticollis
3 = Unlimited ability to watch television but requires extensive measures to control torticollis or is able to view only in nonseated position (e.g. lying down)
4 = Limited ability to watch television because of torticollis
5 = Unable to watch television more than a few minutes because of torticollis

**II-F. Activities Outside the Home (e.g. Shopping, Walking About, Movies, Dining, and Other Recreational Activities)**

0 = No difficulty
1 = Unlimited activities but bothered by torticollis
2 = Unlimited activities but requires simple tricks to accomplish
3 = Accomplishes activities only when accompanied by others because of torticollis
4 = Limited activities outside the home; certain activities impossible or given up because of torticollis
5 = Rarely if ever engages in activities outside the home

**III. Pain Scale (Sum of A-C, Maximum = 20)**

**III-A. Severity of Pain**

*Rate the severity of neck pain due to CD during the last week on a scale of 0 - 10 where a score of 0 represents no pain and 10 represents the most excruciating pain imaginable.*

Best 0 - 10
Worst 0 - 10
Usual 0 - 10
Severity = [Worst + Best + (2 x Usual)]/4

### III-B. Duration of Pain

*Rate the duration of neck pain*
0 = None
1 = Present <10% of the time
2 = Present 10% – 25% of the time
3 = Present 26% – 50% of the time
4 = Present 51% – 75% of the time
5 = Present >76% of the time

### III-C. Disability Due to Pain

0 = No limitation or interference from pain
1 = Pain is quite bothersome but not a source of disability
2 = Pain definitely interferes with some tasks but is not a major contributor to disability
3 = Pain accounts for some (less than half) but not all of disability
4 = Pain is a major source of difficulty with activities; separate from this, head pulling is also a source of some (less than half) disability
5 = Pain is the major source of disability; without it most impaired activities could be performed quite satisfactorily despite the head pulling

# Appendix 2: Clinical Global Impression of Change

## Clinical Global Impression of Change (CGIC)

**Investigator:** Please rate subject's overall improvement or worsening whether or not you think it's due entirely to study medication. Think about subject's condition prior to his (her) last study medication injection and compare it to his (her) condition today; how much has he (she) changed? *(select only one)*

1☐ +3 Very much improved
2☐ +2 Much improved
3☐ +1 Minimally improved
4☐ 0 No change
5☐ -1 Minimally worse
6☐ -2 Much worse
7☐ -3 Very much worse

# Appendix 3: CDIP-58 Questionnaire

Appendix I: The Cervical Dystonia Impact Profile (CDIP-58)

The CDIP-58 includes 58 evaluative items forming eight scales: head and neck symptoms (6 items), pain and discomfort symptoms (5 items), upper limb activities (9 items), walking (9 items) sleep (4 items), annoyance (8 items), mood (7 items), psychosocial functioning (10 items). Eight summary scale scores are generated by summing items and then transformed to a 0-100 scale. High scores indicate worse health.

People like you who have spasmodic torticollis are bothered by different problems. The following questions ask about problem you may have been bothered by during the past 2 weeks.

**1.** During the past 2 weeks, how much were you bothered by each of the following problems? (Please circle the number in box that best describes your situation.)

| | Not at all | A little | Moderately | Quite a bit | Extremely |
|---|---|---|---|---|---|
| a) Uncontrollable movements of your neck preventing your head from being straight? | 1 | 2 | 3 | 4 | 5 |
| b) Twisting of the neck? | 1 | 2 | 3 | 4 | 5 |
| c) Inability to control your head? | 1 | 2 | 3 | 4 | 5 |
| d) Tension in your neck? | 1 | 2 | 3 | 4 | 5 |
| e) Straining in your neck? | 1 | 2 | 3 | 4 | 5 |
| f) Stiffness in your neck? | 1 | 2 | 3 | 4 | 5 |
| g) Aching in your shoulders? | 1 | 2 | 3 | 4 | 5 |
| h) Shoulder pain? | 1 | 2 | 3 | 4 | 5 |
| i) Neck and shoulders being tired? | 1 | 2 | 3 | 4 | 5 |
| j) Tightness in your neck? | 1 | 2 | 3 | 4 | 5 |
| k) Tightness in your shoulders? | 1 | 2 | 3 | 4 | 5 |

2. During the past 2 weeks, has spasmodic torticollis limited your ability to carry out your usual daily activities?

| | Not at all | A little | Moderately | Quite a bit | Extremely |
|---|---|---|---|---|---|
| a) Limits in the type of work or other activities? | 1 | 2 | 3 | 4 | 5 |
| b) Carrying heavy objects? | 1 | 2 | 3 | 4 | 5 |
| c) Carrying light objects? | 1 | 2 | 3 | 4 | 5 |
| d) Heavy household chores? | 1 | 2 | 3 | 4 | 5 |
| e) Light household chores? | 1 | 2 | 3 | 4 | 5 |
| f) Cleaning the house? | 1 | 2 | 3 | 4 | 5 |
| g) Cooking? | 1 | 2 | 3 | 4 | 5 |
| h) Getting tired when doing demanding physical activities? | 1 | 2 | 3 | 4 | 5 |
| i) Getting tired when doing light physical activities? | 1 | 2 | 3 | 4 | 5 |

3. During the past 2 weeks, how much has your spasmodic torticollis:

| | Not at all | A little | Moderately | Quite a bit | Extremely |
|---|---|---|---|---|---|
| a) Limited your ability to walk? | 1 | 2 | 3 | 4 | 5 |
| b) Limited your ability to climb up and down stairs? | 1 | 2 | 3 | 4 | 5 |
| c) Limited how far you are able to walk? | 1 | 2 | 3 | 4 | 5 |
| d) Increased the effort needed for you to walk? | 1 | 2 | 3 | 4 | 5 |
| e) Slowed down your walking? | 1 | 2 | 3 | 4 | 5 |
| f) Affected how smoothly you walk? | 1 | 2 | 3 | 4 | 5 |
| g) Made you concentrate on your walking? | 1 | 2 | 3 | 4 | 5 |
| h) Made you feel unsafe walking up and down stairs? | 1 | 2 | 3 | 4 | 5 |
| i) Made you feel unsteady walking? | 1 | 2 | 3 | 4 | 5 |

©University College London, UK and Peninsula Medical School, Plymouth, UK 2004

©University College London, UK and Peninsula Medical School, Plymouth, UK 2004

4. During the past 2 weeks, how often did you:

| | None of the time | A little of the time | Some of the time | Most of the time | All of the time |
|---|---|---|---|---|---|
| a) Have trouble falling asleep because of the symptoms of your spasmodic torticollis? | 1 | 2 | 3 | 4 | 5 |
| b) Have a restless sleep because of the symptoms of your spasmodic torticollis? | 1 | 2 | 3 | 4 | 5 |
| c) Wake up because of the symptoms of your spasmodic torticollis? | 1 | 2 | 3 | 4 | 5 |
| d) Not get the amount of sleep that you needed because of the symptoms of your spasmodic torticollis? | 1 | 2 | 3 | 4 | 5 |

5. During the past 2 weeks, has spasmodic torticollis limited your ability to carry out your usual social activities?

| | Not at all | A little | Moderately | Quite a bit | Extremely |
|---|---|---|---|---|---|
| a) Enjoyment of social situations? | 1 | 2 | 3 | 4 | 5 |
| b) Socialising with friends of family? | 1 | 2 | 3 | 4 | 5 |

6. During the past 2 weeks, how often has spasmodic torticollis caused you to feel:

| | None of the time | A little of the time | Some of the time | Most of the time | All of the time |
|---|---|---|---|---|---|
| a) Angry? | 1 | 2 | 3 | 4 | 5 |
| b) Annoyed? | 1 | 2 | 3 | 4 | 5 |
| c) Irritated? | 1 | 2 | 3 | 4 | 5 |
| d) Aggravated? | 1 | 2 | 3 | 4 | 5 |
| e) Fed up? | 1 | 2 | 3 | 4 | 5 |
| f) Frustrated? | 1 | 2 | 3 | 4 | 5 |
| g) Stressed? | 1 | 2 | 3 | 4 | 5 |
| h) Impatient? | 1 | 2 | 3 | 4 | 5 |
| i) Upset? | 1 | 2 | 3 | 4 | 5 |
| j) Worried? | 1 | 2 | 3 | 4 | 5 |
| k) Anxious? | 1 | 2 | 3 | 4 | 5 |
| l) Scared? | 1 | 2 | 3 | 4 | 5 |
| m) Fearful? | 1 | 2 | 3 | 4 | 5 |
| n) Depressed? | 1 | 2 | 3 | 4 | 5 |
| o) Down? | 1 | 2 | 3 | 4 | 5 |
| p) More self-conscious in social situations? | 1 | 2 | 3 | 4 | 5 |
| q) Uneasy talking to strangers? | 1 | 2 | 3 | 4 | 5 |
| r) Less relaxed in social situations? | 1 | 2 | 3 | 4 | 5 |
| s) Embarrassed about eating in public (eg café, restaurant)? | 1 | 2 | 3 | 4 | 5 |
| t) Embarrassed going out in public (eg cinema, theatre)? | 1 | 2 | 3 | 4 | 5 |
| u) Everybody is staring at you? | 1 | 2 | 3 | 4 | 5 |
| v) Lack of confidence? | 1 | 2 | 3 | 4 | 5 |
| w) Lack of self-confidence? | 1 | 2 | 3 | 4 | 5 |

©University College London, UK and Peninsula Medical School, Plymouth, UK 2004

# Appendix 4: Patient Global Impression of Change

## Patient's Global Impression of Change (PGIC)

**Patient:** Please rate your overall improvement or worsening whether or not you think it's due entirely to study medication. Think about your condition prior to your last study medication injection and compare it to your condition today; how much have you changed? *(select only one)*

1☐ +3 Very much improved
2☐ +2 Much improved
3☐ +1 Minimally improved
4☐ 0 No change
5☐ -1 Minimally worse
6☐ -2 Much worse
7☐ -3 Very much worse

# Appendix 5: Numeric Rating Scale: Subject Assessment of Pain

## Pain Numeric Rating Scale: Subject Assessment of Pain

To evaluate your pain, we will use a validated Pain Numeric Rating Scale (NRS). The NRS below provides a representation of pain on an 11-point scale from no pain to worst possible pain. Please tick one of the boxes which best describes the intensity of your pain.

### PAIN NUMERIC RATING SCALE (NRS)

How much pain have you had because of your cervical dystonia in the past 24 hours?
Please check appropriate number to indicate the severity of pain.

| No Pain | | | | | | | | | | Worst Possible Pain |
|---|---|---|---|---|---|---|---|---|---|---|
| 0 ☐ | 1 ☐ | 2 ☐ | 3 ☐ | 4 ☐ | 5 ☐ | 6 ☐ | 7 ☐ | 8 ☐ | 9 ☐ | 10 ☐ |

# Appendix 6: Brief Pain Inventory Short Form

## Brief Pain Inventory (Short Form)

**Circle the one number that describes how, during the past 2 weeks, cervical dystonia pain has interfered with your:**

**General Activity**

| 0 | 1 | 2 | 3 | 4 | 5 | 6 | 7 | 8 | 9 | 10 |
|---|---|---|---|---|---|---|---|---|---|---|
| Does not interfere | | | | | | | | | | Completely Interferes |

**Mood**

| 0 | 1 | 2 | 3 | 4 | 5 | 6 | 7 | 8 | 9 | 10 |
|---|---|---|---|---|---|---|---|---|---|---|
| Does not interfere | | | | | | | | | | Completely Interferes |

**Walking Ability**

| 0 | 1 | 2 | 3 | 4 | 5 | 6 | 7 | 8 | 9 | 10 |
|---|---|---|---|---|---|---|---|---|---|---|
| Does not interfere | | | | | | | | | | Completely Interferes |

**Normal Work (includes both work outside the home and housework)**

| 0 | 1 | 2 | 3 | 4 | 5 | 6 | 7 | 8 | 9 | 10 |
|---|---|---|---|---|---|---|---|---|---|---|
| Does not interfere | | | | | | | | | | Completely Interferes |

**Relations with other people**

| 0 | 1 | 2 | 3 | 4 | 5 | 6 | 7 | 8 | 9 | 10 |
|---|---|---|---|---|---|---|---|---|---|---|
| Does not interfere | | | | | | | | | | Completely Interferes |

**Sleep**

| 0 | 1 | 2 | 3 | 4 | 5 | 6 | 7 | 8 | 9 | 10 |
|---|---|---|---|---|---|---|---|---|---|---|
| Does not interfere | | | | | | | | | | Completely Interferes |

**Enjoyment of life**

| 0 | 1 | 2 | 3 | 4 | 5 | 6 | 7 | 8 | 9 | 10 |
|---|---|---|---|---|---|---|---|---|---|---|
| Does not interfere | | | | | | | | | | Completely Interferes |

Copyright 1991 Charles S. Cleeland, PhD
Pain Research Group
All rights reserved

# Appendix 7: Patient Health Questionnaire (PHQ)-9

## Patient Health Questionnaire (PHQ)-9

Fill in the boxes with pen or pencil to mark your answers.

(a) Over the last 2 weeks, how often have you been bothered by any of the following problems?

| | | **Not At All** | **Several Days** | **More Than Half the Days** | **Nearly Every Day** |
|---|---|---|---|---|---|
| | | 0 | 1 | 2 | 3 |
| 1 | Little interest or pleasure in doing things. | □ | □ | □ | □ |
| 2 | Feeling down, depressed or hopeless. | □ | □ | □ | □ |
| 3 | Trouble falling/staying asleep, sleeping too much. | □ | □ | □ | □ |
| 4 | Feeling tired or having little energy. | □ | □ | □ | □ |
| 5 | Poor appetite or overeating. | □ | □ | □ | □ |
| 6 | Feeling bad about yourself, or that you are a failure or have let yourself or your family down. | □ | □ | □ | □ |
| 7 | Trouble concentrating on things, such as reading the newspaper or watching television | □ | □ | □ | □ |
| 8 | Moving or speaking so slowly that other people could have noticed. Or the opposite, being so fidgety or restless that you have being moving around a lot more than usual. | □ | □ | □ | □ |
| 9 | Thoughts that you would be better off dead or of hurting yourself in some way. | □ | □ | □ | □ |
| | Total Score = | | | | |

b) If you have been bothered by any of the nine problems listed above, please answer the following:
How difficult have these problems made it for you to do your work, take care of things at home, or get along with other people?

| Not difficult at all | Somewhat difficult | Very difficult | Extremely difficult |
|---|---|---|---|
| □ | □ | □ | □ |

# Appendix 8: Modified TSQM-9

## Modified TSQM-9
## Abbreviated Treatment Satisfaction Questionnaire for Medication

**Instructions**: Please take some time to think about your level of satisfaction or dissatisfaction with the medication (Dysport) you are taking in this clinical study. We are interested in your evaluation of the effectiveness and convenience of the medication (Dysport) *over the last two to three weeks, or since you last used it.* For each question, please place a single check mark next to the response that most closely corresponds to your own experiences.

1. How satisfied or dissatisfied are you with the ability of the medication to prevent or treat your condition?

□1 Extremely Dissatisfied
□2 Very Dissatisfied
□3 Dissatisfied
□4 Somewhat Satisfied
□5 Satisfied
□6 Very Satisfied
□7 Extremely Satisfied

2. How satisfied or dissatisfied are you with the way the medication relieves your symptoms?

□1 Extremely Dissatisfied
□2 Very Dissatisfied
□3 Dissatisfied
□4 Somewhat Satisfied
□5 Satisfied
□6 Very Satisfied
□7 Extremely Satisfied

3. How satisfied or dissatisfied are you with the amount of time it takes the medication to start working?

□1 Extremely Dissatisfied
□2 Very Dissatisfied
□3 Dissatisfied
□4 Somewhat Satisfied
□5 Satisfied
□6 Very Satisfied
□7 Extremely Satisfied

4. Overall, how confident are you that taking this medication is a good thing for you?

□1 Not at All Confident
□2 A Little Confident
□3 Somewhat Confident
□4 Very Confident
□5 Extremely Confident

5. How certain are you that the good things about your medication outweigh the bad things?

□1 Not at All Certain
□2 A Little Certain
□3 Somewhat Certain
□4 Very Certain
□5 Extremely Certain

6. Taking all things into account, how satisfied or dissatisfied are you with this medication?

□1 Extremely Dissatisfied
□2 Very Dissatisfied
□3 Dissatisfied
□4 Somewhat Satisfied
□5 Satisfied
□6 Very Satisfied
□7 Extremely Satisfied

# Appendix 9: C-SSRS - Already Enrolled Subjects

# COLUMBIA-SUICIDE SEVERITY RATING SCALE (C-SSRS)

Already Enrolled Subjects

Version 1/14/09

*Posner, K.; Brent, D.; Lucas, C.; Gould, M.; Stanley, B.; Brown, G.; Fisher, P.; Zelazny, J.; Burke, A.; Oquendo, M.; Mann, J.*

*Disclaimer:*
*This scale is intended to be used by individuals who have received training in its administration. The questions contained in the Columbia-Suicide Severity Rating Scale are suggested probes. Ultimately, the determination of the presence of suicidal ideation or behavior depends on the judgment of the individual administering the scale.*

*Definitions of behavioral suicidal events in this scale are based on those used in* <u>The Columbia Suicide History Form</u>*, developed by John Mann, MD and Maria Oquendo, MD, Conte Center for the Neuroscience of Mental Disorders (CCNMD), New York State Psychiatric Institute, 1051 Riverside Drive, New York, NY, 10032. (Oquendo M. A., Halberstam B. & Mann J. J., Risk factors for suicidal behavior: utility and limitations of research instruments. In M.B. First [Ed.]* Standardized Evaluation in Clinical Practice*, pp. 103 -130, 2003.)*

*For reprints of the C-SSRS contact Kelly Posner, Ph.D., New York State Psychiatric Institute, 1051 Riverside Drive, New York, New York, 10032; inquiries and training requirements contact posnerk@nyspi.columbia.edu*

© 2008 The Research Foundation for Mental Hygiene, Inc.

| ***SUICIDAL IDEATION*** | | |
|---|---|---|
| *Ask questions 1 and 2. If both are negative, proceed to "Suicidal Behavior" section. If the answer to question 2 is "yes", ask questions 3, 4 and 5. If the answer to question 1 and/or 2 is "yes", complete "Intensity of Ideation" section below.* | **Prior to Study Entry: Time He/She Felt Most Suicidal** | **Since Study Start:** |
| **1. Wish to be Dead**<br>Subject endorses thoughts about a wish to be dead or not alive anymore, or wish to fall asleep and not wake up.<br>***Have you wished you were dead or wished you could go to sleep and not wake up?***<br><br>If yes, describe: | **Yes** ☐ **No** ☐ | **Yes** ☐ **No** ☐ |
| **2. Non-Specific Active Suicidal Thoughts**<br>General non-specific thoughts of wanting to end one's life/commit suicide (e.g., *"I've thought about killing myself"*) without thoughts of ways to kill oneself/associated methods, intent, or plan during the assessment period.<br>***Have you actually had any thoughts of killing yourself?***<br><br>If yes, describe: | **Yes** ☐ **No** ☐ | **Yes** ☐ **No** ☐ |
| **3. Active Suicidal Ideation with Any Methods (Not Plan) without Intent to Act**<br>Subject endorses thoughts of suicide and has thought of at least one method during the assessment period. This is different than a specific plan with time, place or method details worked out (e.g., thought of method to kill self but not a specific plan). Includes person who would say, *"I thought about taking an overdose but I never made a specific plan as to when, where or how I would actually do it…and I would never go through with it."*<br>***Have you been thinking about how you might do this?***<br><br>If yes, describe: | **Yes** ☐ **No** ☐ | **Yes** ☐ **No** ☐ |
| **4. Active Suicidal Ideation with Some Intent to Act, without Specific Plan**<br>Active suicidal thoughts of killing oneself and subject reports having some intent to act on such thoughts, as opposed to *"I have the thoughts but I definitely will not do anything about them."*<br>***Have you had these thoughts and had some intention of acting on them?***<br><br>If yes, describe: | **Yes** ☐ **No** ☐ | **Yes** ☐ **No** ☐ |
| **5. Active Suicidal Ideation with Specific Plan and Intent**<br>Thoughts of killing oneself with details of plan fully or partially worked out and subject has some intent to carry it out.<br>***Have you started to work out or worked out the details of how to kill yourself? Do you intend to carry out this plan?***<br><br>If yes, describe: | **Yes** ☐ **No** ☐ | **Yes** ☐ **No** ☐ |
| ***INTENSITY OF IDEATION*** | | |
| *The following features should be rated with respect to the most severe type of ideation (i.e., 1-5 from above, with 1 being the least severe and 5 being the most severe). For prior to study entry, ask about time he/she was feeling the most suicidal.*<br><br>Prior to Study Entry –<br>***Most Severe Ideation:*** ______<br>____________________<br>***Type # (1-5)*** ***Description of Ideation***<br><br>Since Study Start –<br>***Most Severe Ideation:*** ______<br>____________________<br>***Type # (1-5)*** ***Description of Ideation*** | Most Severe | Most Severe |
| **Frequency**<br>***How many times have you had these thoughts?***<br>(1) Less than once a week (2) Once a week (3) 2-5 times in week<br>(4) Daily or almost daily (5) Many times each day | ____ | ____ |

| | | |
|---|---|---|
| **Duration**<br>***When you have the thoughts how long do they last?***<br>(1) Fleeting - few seconds or minutes (4) 4-8 hours/most of day<br>(2) Less than 1 hour/some of the time (5) More than 8 hours/persistent or continuous<br>(3) 1-4 hours/a lot of time | —— | —— |
| **Controllability**<br>***Could/can you stop thinking about killing yourself or wanting to die if you want to?***<br>(1) Easily able to control thoughts (4) Can control thoughts with a lot of difficulty<br>(2) Can control thoughts with little difficulty (5) Unable to control thoughts<br>(3) Can control thoughts with some difficulty (0) Does not attempt to control thoughts | —— | —— |
| **Deterrents**<br>***Are there things - anyone or anything (e.g., family, religion, pain of death) - that stopped you from wanting to die or acting on thoughts of committing suicide?***<br>(1) Deterrents definitely stopped you from attempting suicide (4) Deterrents most likely did not stop you<br>(5) Deterrents definitely did not stop you<br>(2) Deterrents probably stopped you (0) Does not apply<br>(3) Uncertain that deterrents stopped you | —— | —— |
| **Reasons for Ideation**<br>***What sort of reasons did you have for thinking about wanting to die or killing yourself? Was it to end the pain or stop the way you were feeling (in other words you couldn't go on living with this pain or how you were feeling) or was it to get attention, revenge or a reaction from others? Or both?***<br>(1) Completely to get attention, revenge or a reaction from others (4) Mostly to end or stop the pain (you couldn't go on living with the pain or how you were feeling)<br>(2) Mostly to get attention, revenge or a reaction from others (5) Completely to end or stop the pain (you couldn't go on living with the pain or how you were feeling)<br>(3) Equally to get attention, revenge or a reaction from others and to end/stop the pain (0) Does not apply | —— | —— |

Version 1/14/09

| ***SUICIDAL BEHAVIOR*** *(Check all that apply, so long as these are separate events; must ask about all types)* | **Prior to Study Entry** | **Since Study Start** |
|---|---|---|
| **Actual Attempt:**<br>A potentially self-injurious act committed with at least some wish to die, *as a result of act.* Behavior was in part thought of as method to kill oneself. Intent does not have to be 100%. If there is ***any*** intent/desire to die associated with the act, then it can be considered an actual suicide attempt. ***There does not have to be any injury or harm***, just the potential for injury or harm. If person pulls trigger while gun is in mouth but gun is broken so no injury results, this is considered an attempt.<br>Inferring Intent: Even if an individual denies intent/wish to die, it may be inferred clinically from the behavior or circumstances. For example, a highly lethal act that is clearly not an accident so no other intent but suicide can be inferred (e.g., gunshot to head, jumping from window of a high floor/story). Also, if someone denies intent to die, but they thought that what they did could be lethal, intent may be inferred.<br>***Have you made a suicide attempt?***<br>***Have you done anything to harm yourself?***<br>***Have you done anything dangerous where you could have died?***<br>***What did you do?***<br>***Did you______ as a way to end your life?***<br>***Did you want to die (even a little) when you_____?***<br>***Were you trying to end your life when you _____?***<br>***Or Did you think it was possible you could have died from_____?***<br>***Or did you do it purely for other reasons / without ANY intention of killing yourself (like to relieve stress, feel better, get sympathy, or get something else to happen)?***<br>*(*Self-Injurious Behavior without suicidal intent)<br>If yes, describe: | **Yes** **No**<br>☐ ☐<br><br>Total # of Attempts<br>______ | **Yes** **No**<br>☐ ☐<br><br>Total # of Attempts<br>______ |
| **Has subject engaged in Non-Suicidal Self-Injurious Behavior?** | **Yes** **No**<br>☐ ☐ | **Yes** **No**<br>☐ ☐ |
| **Interrupted Attempt:**<br>When the person is interrupted (by an outside circumstance) from starting the potentially self-injurious act *(if not for that, actual attempt would have occurred).*<br>Overdose: Person has pills in hand but is stopped from ingesting. Once they ingest any pills, this becomes an attempt rather than an interrupted attempt. Shooting: Person has gun pointed toward self, gun is taken away by someone else, or is somehow prevented from pulling trigger. Once they pull the trigger, even if the gun fails to fire, it is an attempt. Jumping: Person is poised to jump, is grabbed and taken down from ledge. Hanging: Person has noose around neck but has not yet started to hang - is stopped from doing so.<br>***Has there been a time when you started to do something to end your life but someone or something stopped you before you actually did anything?***<br>If yes, describe: | **Yes** **No**<br>☐ ☐<br><br>Total # of interrupted<br>______ | **Yes** **No**<br>☐ ☐<br><br>Total # of interrupted<br>______ |
| **Aborted Attempt:**<br>When person begins to take steps toward making a suicide attempt, but stops themselves before they actually have engaged in any self-destructive behavior. Examples are similar to interrupted attempts, except that the individual stops him/herself, instead of being stopped by something else.<br>***Has there been a time when you started to do something to try to end your life but you stopped yourself before you actually did anything?***<br>If yes, describe: | **Yes** **No**<br>☐ ☐<br><br>Total # of aborted<br>______ | **Yes** **No**<br>☐ ☐<br><br>Total # of aborted<br>______ |
| **Preparatory Acts or Behavior:**<br>Acts or preparation towards imminently making a suicide attempt. This can include anything beyond a verbalization or thought, such as assembling a specific method (e.g., buying pills, purchasing a gun) or preparing for one's death by suicide (e.g., giving things away, writing a suicide note).<br>***Have you taken any steps towards making a suicide attempt or preparing to kill yourself (such as collecting pills, getting a gun, giving valuables away or writing a suicide note)?***<br>If yes, describe: | **Yes** **No**<br>☐ ☐ | **Yes** **No**<br>☐ ☐ |
| **Suicidal Behavior:**<br>Suicidal behavior was present during the assessment period? | **Yes** **No**<br>☐ ☐ | **Yes** **No**<br>☐ ☐ |
| **Completed Suicide:** | **Yes** **No**<br>☐ ☐ | **Yes** **No**<br>☐ ☐ |

| ***Answer for Actual Attempts Only*** | Most Recent Attempt Date: | Most Lethal Attempt Date: | Initial/First Attempt Date: |
|---|---|---|---|
| **Actual Lethality/Medical Damage:**<br>0. No physical damage or very minor physical damage (e.g., surface scratches).<br>1. Minor physical damage (e.g., lethargic speech; first-degree burns; mild bleeding; sprains).<br>2. Moderate physical damage; medical attention needed (e.g., conscious but sleepy, somewhat responsive; second-degree burns; bleeding of major vessel).<br>3. Moderately severe physical damage; *medical* hospitalization and likely intensive care required (e.g., comatose with reflexes intact; third-degree burns less than 20% of body; extensive blood loss but can recover; major fractures).<br>4. Severe physical damage; *medical* hospitalization with intensive care required (e.g., comatose without reflexes; third-degree burns over 20% of body; extensive blood loss with unstable vital signs; major damage to a vital area).<br>5. Death | *Enter Code*<br>______ | *Enter Code*<br>______ | *Enter Code*<br>______ |
| **Potential Lethality: Only Answer if Actual Lethality=0**<br>Likely lethality of actual attempt if no medical damage (the following examples, while having no actual medical damage, had potential for very serious lethality: put gun in mouth and pulled the trigger but gun fails to fire so no medical damage; laying on train tracks with oncoming train but pulled away before run over).<br>0 = Behavior not likely to result in injury<br>1 = Behavior likely to result in injury but not likely to cause death<br>2 = Behavior likely to result in death despite available medical care | *Enter Code*<br>______ | *Enter Code*<br>______ | *Enter Code*<br>______ |

# Appendix 10: C-SSRS - Baseline/Screening

# COLUMBIA-SUICIDE SEVERITY RATING SCALE (C-SSRS)

Baseline/Screening Version

Version 1/14/09

*Posner, K.; Brent, D.; Lucas, C.; Gould, M.; Stanley, B.; Brown, G.; Fisher, P.; Zelazny, J.; Burke, A.; Oquendo, M.; Mann, J.*

*Disclaimer:*
*This scale is intended to be used by individuals who have received training in its administration. The questions contained in the Columbia-Suicide Severity Rating Scale are suggested probes. Ultimately, the determination of the presence of suicidal ideation or behavior depends on the judgment of the individual administering the scale.*

*Definitions of behavioral suicidal events in this scale are based on those used in The Columbia Suicide History Form, developed by John Mann, MD and Maria Oquendo, MD, Conte Center for the Neuroscience of Mental Disorders (CCNMD), New York State Psychiatric Institute, 1051 Riverside Drive, New York, NY, 10032. (Oquendo M. A., Halberstam B. & Mann J. J., Risk factors for suicidal behavior: utility and limitations of research instruments. In M.B. First [Ed.]* Standardized Evaluation in Clinical Practice*, pp. 103 -130, 2003.)*

*For reprints of the C-SSRS contact Kelly Posner, Ph.D., New York State Psychiatric Institute, 1051 Riverside Drive, New York, New York, 10032; inquiries and training requirements contact posnerk@nyspi.columbia.edu*

© 2008 The Research Foundation for Mental Hygiene, Inc.

| *SUICIDAL IDEATION* | | |
|---|---|---|
| *Ask questions 1 and 2. If both are negative, proceed to "Suicidal Behavior" section. If the answer to question 2 is "yes", ask questions 3, 4 and 5. If the answer to question 1 and/or 2 is "yes", complete "Intensity of Ideation" section below.* | **Lifetime: Time He/She Felt Most Suicidal** | **Past __ Months** |
| **1. Wish to be Dead**<br>Subject endorses thoughts about a wish to be dead or not alive anymore, or wish to fall asleep and not wake up.<br>***Have you wished you were dead or wished you could go to sleep and not wake up?***<br><br>If yes, describe: | **Yes** ☐ **No** ☐ | **Yes** ☐ **No** ☐ |
| **2. Non-Specific Active Suicidal Thoughts**<br>General non-specific thoughts of wanting to end one's life/commit suicide (e.g., *"I've thought about killing myself"*) without thoughts of ways to kill oneself/associated methods, intent, or plan during the assessment period.<br>***Have you actually had any thoughts of killing yourself?***<br><br>If yes, describe: | **Yes** ☐ **No** ☐ | **Yes** ☐ **No** ☐ |
| **3. Active Suicidal Ideation with Any Methods (Not Plan) without Intent to Act**<br>Subject endorses thoughts of suicide and has thought of at least one method during the assessment period. This is different than a specific plan with time, place or method details worked out (e.g. thought of method to kill self but not a specific plan). Includes person who would say, *"I thought about taking an overdose but I never made a specific plan as to when, where or how I would actually do it…and I would never go through with it."*<br>***Have you been thinking about how you might do this?***<br><br>If yes, describe: | **Yes** ☐ **No** ☐ | **Yes** ☐ **No** ☐ |
| **4. Active Suicidal Ideation with Some Intent to Act, without Specific Plan**<br>Active suicidal thoughts of killing oneself and subject reports having <u>some intent to act on such thoughts</u>, as opposed to *"I have the thoughts but I definitely will not do anything about them."*<br>***Have you had these thoughts and had some intention of acting on them?***<br><br>If yes, describe: | **Yes** ☐ **No** ☐ | **Yes** ☐ **No** ☐ |
| **5. Active Suicidal Ideation with Specific Plan and Intent**<br>Thoughts of killing oneself with details of plan fully or partially worked out and subject has some intent to carry it out.<br>***Have you started to work out or worked out the details of how to kill yourself? Do you intend to carry out this plan?***<br><br>If yes, describe: | **Yes** ☐ **No** ☐ | **Yes** ☐ **No** ☐ |
| *INTENSITY OF IDEATION* | | |
| *The following features should be rated with respect to the most severe type of ideation (i.e., 1-5 from above, with 1 being the least severe and 5 being the most severe). Ask about time he/she was feeling the most suicidal.*<br><br><u>Lifetime</u> -<br>***Most Severe Ideation:*** _______ ________________________<br>***Type # (1-5)*** ***Description of Ideation***<br><br><u>Past X Months</u> –<br>***Most Severe Ideation:*** _______ ________________________<br>***Type # (1-5)*** ***Description of Ideation*** | Most Severe | Most Severe |
| **Frequency**<br>***How many times have you had these thoughts?***<br>(1) Less than once a week (2) Once a week (3) 2-5 times in week (4) Daily or almost daily (5) Many times each day | ____ | ____ |

| | | |
|---|---|---|
| **Duration**<br>***When you have the thoughts how long do they last?***<br>(1) Fleeting - few seconds or minutes<br>(2) Less than 1 hour/some of the time<br>(3) 1-4 hours/a lot of time<br>(4) 4-8 hours/most of day<br>(5) More than 8 hours/persistent or continuous | ____ | ____ |
| **Controllability**<br>***Could/can you stop thinking about killing yourself or wanting to die if you want to?***<br>(1) Easily able to control thoughts<br>(2) Can control thoughts with little difficulty<br>(3) Can control thoughts with some difficulty<br>(4) Can control thoughts with a lot of difficulty<br>(5) Unable to control thoughts<br>(0) Does not attempt to control thoughts | ____ | ____ |
| **Deterrents**<br>***Are there things - anyone or anything (e.g., family, religion, pain of death) - that stopped you from wanting to die or acting on thoughts of committing suicide?***<br>(1) Deterrents definitely stopped you from attempting suicide<br>(2) Deterrents probably stopped you<br>(3) Uncertain that deterrents stopped you<br>(4) Deterrents most likely did not stop you<br>(5) Deterrents definitely did not stop you<br>(0) Does not apply | ____ | ____ |
| **Reasons for Ideation**<br>***What sort of reasons did you have for thinking about wanting to die or killing yourself? Was it to end the pain or stop the way you were feeling (in other words you couldn't go on living with this pain or how you were feeling) or was it to get attention, revenge or a reaction from others? Or both?***<br>(1) Completely to get attention, revenge or a reaction from others<br>(2) Mostly to get attention, revenge or a reaction from others<br>(3) Equally to get attention, revenge or a reaction from others and to end/stop the pain<br>(4) Mostly to end or stop the pain (you couldn't go on living with the pain or how you were feeling)<br>(5) Completely to end or stop the pain (you couldn't go on living with the pain or how you were feeling)<br>(0) Does not apply | ____ | ____ |

Version 1/14/09

| ***SUICIDAL BEHAVIOR***<br>*(Check all that apply, so long as these are separate events; must ask about all types)* | **Lifetime** | **Past __ Years** |
|---|---|---|
| **Actual Attempt:**<br>A potentially self-injurious act committed with at least some wish to die, *as a result of act.* Behavior was in part thought of as method to kill oneself. Intent does not have to be 100%. If there is ***any*** intent/desire to die associated with the act, then it can be considered an actual suicide attempt. ***There does not have to be any injury or harm***, just the potential for injury or harm. If person pulls trigger while gun is in mouth but gun is broken so no injury results, this is considered an attempt.<br>Inferring Intent: Even if an individual denies intent/wish to die, it may be inferred clinically from the behavior or circumstances. For example, a highly lethal act that is clearly not an accident so no other intent but suicide can be inferred (e.g., gunshot to head, jumping from window of a high floor/story). Also, if someone denies intent to die, but they thought that what they did could be lethal, intent may be inferred.<br>***Have you made a suicide attempt?***<br>***Have you done anything to harm yourself?***<br>***Have you done anything dangerous where you could have died?***<br>***What did you do?***<br>***Did you______ as a way to end your life?***<br>***Did you want to die (even a little) when you_____?***<br>***Were you trying to end your life when you _____?***<br>***Or Did you think it was possible you could have died from_____?***<br>***Or did you do it purely for other reasons / without ANY intention of killing yourself (like to relieve stress, feel better, get sympathy, or get something else to happen)?*** *(*Self-Injurious Behavior without suicidal intent)<br>If yes, describe: | **Yes** **No**<br>☐ ☐<br><br>Total # of Attempts<br>______ | **Yes** **No**<br>☐ ☐<br><br>Total # of Attempts<br>______ |
| **Has subject engaged in Non-Suicidal Self-Injurious Behavior?** | **Yes** **No**<br>☐ ☐ | **Yes** **No**<br>☐ ☐ |

| | | |
|---|---|---|
| **Interrupted Attempt:**<br>When the person is interrupted (by an outside circumstance) from starting the potentially self-injurious act *(if not for that, actual attempt would have occurred).*<br>Overdose: Person has pills in hand but is stopped from ingesting. Once they ingest any pills, this becomes an attempt rather than an interrupted attempt. Shooting: Person has gun pointed toward self, gun is taken away by someone else, or is somehow prevented from pulling trigger. Once they pull the trigger, even if the gun fails to fire, it is an attempt. Jumping: Person is poised to jump, is grabbed and taken down from ledge. Hanging: Person has noose around neck but has not yet started to hang - is stopped from doing so.<br>***Has there been a time when you started to do something to end your life but someone or something stopped you before you actually did anything?***<br>If yes, describe: | **Yes** **No**<br>☐ ☐<br>Total # of interrupted<br>______ | **Yes** **No**<br>☐ ☐<br>Total # of interrupted<br>______ |
| **Aborted Attempt:**<br>When person begins to take steps toward making a suicide attempt, but stops themselves before they actually have engaged in any self-destructive behavior. Examples are similar to interrupted attempts, except that the individual stops him/herself, instead of being stopped by something else.<br>***Has there been a time when you started to do something to try to end your life but you stopped yourself before you actually did anything?***<br>If yes, describe: | **Yes** **No**<br>☐ ☐<br>Total # of aborted<br>______ | **Yes** **No**<br>☐ ☐<br>Total # of aborted<br>______ |
| **Preparatory Acts or Behavior:**<br>Acts or preparation towards imminently making a suicide attempt. This can include anything beyond a verbalization or thought, such as assembling a specific method (e.g., buying pills, purchasing a gun) or preparing for one's death by suicide (e.g., giving things away, writing a suicide note).<br>***Have you taken any steps towards making a suicide attempt or preparing to kill yourself (such as collecting pills, getting a gun, giving valuables away or writing a suicide note)?***<br>If yes, describe: | **Yes** **No**<br>☐ ☐ | **Yes** **No**<br>☐ ☐ |
| **Suicidal Behavior:**<br>Suicidal behavior was present during the assessment period? | **Yes** **No**<br>☐ ☐ | **Yes** **No**<br>☐ ☐ |

| ***Answer for Actual Attempts Only*** | Most Recent Attempt Date: | Most Lethal Attempt Date: | Initial/First Attempt Date: |
|---|---|---|---|
| **Actual Lethality/Medical Damage:**<br>0. No physical damage or very minor physical damage (e.g., surface scratches).<br>1. Minor physical damage (e.g., lethargic speech; first-degree burns; mild bleeding; sprains).<br>2. Moderate physical damage; medical attention needed (e.g., conscious but sleepy, somewhat responsive; second-degree burns; bleeding of major vessel).<br>3. Moderately severe physical damage; *medical* hospitalization and likely intensive care required (e.g., comatose with reflexes intact; third-degree burns less than 20% of body; extensive blood loss but can recover; major fractures).<br>4. Severe physical damage; *medical* hospitalization with intensive care required (e.g., comatose without reflexes; third-degree burns over 20% of body; extensive blood loss with unstable vital signs; major damage to a vital area).<br>5. Death | *Enter Code*<br>______ | *Enter Code*<br>______ | *Enter Code*<br>______ |
| **Potential Lethality: Only Answer if Actual Lethality=0**<br>Likely lethality of actual attempt if no medical damage (the following examples, while having no actual medical damage, had potential for very serious lethality: put gun in mouth and pulled the trigger but gun fails to fire so no medical damage; laying on train tracks with oncoming train but pulled away before run over).<br>0 = Behavior not likely to result in injury<br>1 = Behavior likely to result in injury but not likely to cause death<br>2 = Behavior likely to result in death despite available medical care | *Enter Code*<br>______ | *Enter Code*<br>______ | *Enter Code*<br>______ |

# Appendix 11: C-SSRS – Since Last Visit

# COLUMBIA-SUICIDE SEVERITY RATING SCALE (C-SSRS)

Since Last Visit

Version 1/14/09

*Posner, K.; Brent, D.; Lucas, C.; Gould, M.; Stanley, B.; Brown, G.; Fisher, P.; Zelazny, J.; Burke, A.; Oquendo, M.; Mann, J.*

*Disclaimer:*
*This scale is intended to be used by individuals who have received training in its administration. The questions contained in the Columbia-Suicide Severity Rating Scale are suggested probes. Ultimately, the determination of the presence of suicidal ideation or behavior depends on the judgment of the individual administering the scale.*

*Definitions of behavioral suicidal events in this scale are based on those used in The Columbia Suicide History Form, developed by John Mann, MD and Maria Oquendo, MD, Conte Center for the Neuroscience of Mental Disorders (CCNMD), New York State Psychiatric Institute, 1051 Riverside Drive, New York, NY, 10032. (Oquendo M. A., Halberstam B. & Mann J. J., Risk factors for suicidal behavior: utility and limitations of research instruments. In M.B. First [Ed.]* Standardized Evaluation in Clinical Practice*, pp. 103 -130, 2003.)*

*For reprints of the C-SSRS contact Kelly Posner, Ph.D., New York State Psychiatric Institute, 1051 Riverside Drive, New York, New York, 10032; inquiries and training requirements contact posnerk@nyspi.columbia.edu*

© 2008 The Research Foundation for Mental Hygiene, Inc.

| ***SUICIDAL IDEATION*** | |
|---|---|
| *Ask questions 1 and 2. If both are negative, proceed to "Suicidal Behavior" section. If the answer to question 2 is "yes", ask questions 3, 4 and 5. If the answer to question 1 and/or 2 is "yes", complete "Intensity of Ideation" section below.* | Since Last Visit |
| **1. Wish to be Dead**<br>Subject endorses thoughts about a wish to be dead or not alive anymore, or wish to fall asleep and not wake up.<br>***Have you wished you were dead or wished you could go to sleep and not wake up?***<br><br>If yes, describe: | **Yes** **No**<br>☐ ☐ |
| **2. Non-Specific Active Suicidal Thoughts**<br>General, non-specific thoughts of wanting to end one's life/commit suicide (e.g., *"I've thought about killing myself"*) without thoughts of ways to kill oneself/associated methods, intent, or plan during the assessment period.<br>***Have you actually had any thoughts of killing yourself?***<br><br>If yes, describe: | **Yes** **No**<br>☐ ☐ |
| **3. Active Suicidal Ideation with Any Methods (Not Plan) without Intent to Act**<br>Subject endorses thoughts of suicide and has thought of at least one method during the assessment period. This is different than a specific plan with time, place or method details worked out (e.g., thought of method to kill self but not a specific plan). Includes person who would say, *"I thought about taking an overdose but I never made a specific plan as to when, where or how I would actually do it…and I would never go through with it."*<br>***Have you been thinking about how you might do this?***<br><br>If yes, describe: | **Yes** **No**<br>☐ ☐ |
| **4. Active Suicidal Ideation with Some Intent to Act, without Specific Plan**<br>Active suicidal thoughts of killing oneself and subject reports having some intent to act on such thoughts, as opposed to *"I have the thoughts but I definitely will not do anything about them."*<br>***Have you had these thoughts and had some intention of acting on them?***<br><br>If yes, describe: | **Yes** **No**<br>☐ ☐ |
| **5. Active Suicidal Ideation with Specific Plan and Intent**<br>Thoughts of killing oneself with details of plan fully or partially worked out and subject has some intent to carry it out.<br>***Have you started to work out or worked out the details of how to kill yourself? Do you intend to carry out this plan?***<br><br>If yes, describe: | **Yes** **No**<br>☐ ☐ |
| ***INTENSITY OF IDEATION*** | |
| *The following features should be rated with respect to the most severe type of ideation (i.e., 1-5 from above, with 1 being the least severe and 5 being the most severe).*<br><br>***Most Severe Ideation:*** _____ ______________________<br>***Type # (1-5)*** ***Description of Ideation*** | Most Severe |
| **Frequency**<br>***How many times have you had these thoughts?***<br>(1) Less than once a week (2) Once a week (3) 2-5 times in week (4) Daily or almost daily<br>(5) Many times each day | ____ |
| **Duration**<br>***When you have the thoughts, how long do they last?***<br>(1) Fleeting - few seconds or minutes<br>(2) Less than 1 hour/some of the time<br>(3) 1-4 hours/a lot of time<br>(4) 4-8 hours/most of day<br>(5) More than 8 hours/persistent or continuous | ____ |

| | |
|---|---|
| **Controllability**<br>***Could/can you stop thinking about killing yourself or wanting to die if you want to?***<br>(1) Easily able to control thoughts<br>(2) Can control thoughts with little difficulty<br>(3) Can control thoughts with some difficulty<br>(4) Can control thoughts with a lot of difficulty<br>(5) Unable to control thoughts<br>(0) Does not attempt to control thoughts | ____ |
| **Deterrents**<br>***Are there things - anyone or anything (e.g., family, religion, pain of death) - that stopped you from wanting to die or acting on thoughts of committing suicide?***<br>(1) Deterrents definitely stopped you from attempting suicide<br>(2) Deterrents probably stopped you<br>(3) Uncertain that deterrents stopped you<br>(4) Deterrents most likely did not stop you<br>(5) Deterrents definitely did not stop you<br>(0) Does not apply | ____ |
| **Reasons for Ideation**<br>***What sort of reasons did you have for thinking about wanting to die or killing yourself? Was it to end the pain or stop the way you were feeling (in other words you couldn't go on living with this pain or how you were feeling) or was it to get attention, revenge or a reaction from others? Or both?***<br>(1) Completely to get attention, revenge or a reaction from others<br>(2) Mostly to get attention, revenge or a reaction from others<br>(3) Equally to get attention, revenge or a reaction from others and to end/stop the pain<br>(4) Mostly to end or stop the pain (you couldn't go on living with the pain or how you were feeling)<br>(5) Completely to end or stop the pain (you couldn't go on living with the pain or how you were feeling)<br>(0) Does not apply | ____ |

Version 1/14/09

| ***SUICIDAL BEHAVIOR***<br>*(Check all that apply, so long as these are separate events; must ask about all types)* | Since Last Visit |
|---|---|
| **Actual Attempt:**<br>A potentially self-injurious act committed with at least some wish to die, *as a result of act*. Behavior was in part thought of as method to kill oneself. Intent does not have to be 100%. If there is ***any*** intent/desire to die associated with the act, then it can be considered an actual suicide attempt. ***There does not have to be any injury or harm***, just the potential for injury or harm. If person pulls trigger while gun is in mouth but gun is broken so no injury results, this is considered an attempt.<br>Inferring Intent: Even if an individual denies intent/wish to die, it may be inferred clinically from the behavior or circumstances. For example, a highly lethal act that is clearly not an accident so no other intent but suicide can be inferred (e.g., gunshot to head, jumping from window of a high floor/story). Also, if someone denies intent to die, but they thought that what they did could be lethal, intent may be inferred.<br>***Have you made a suicide attempt?***<br>***Have you done anything to harm yourself?***<br>***Have you done anything dangerous where you could have died?***<br>***What did you do?***<br>***Did you______ as a way to end your life?***<br>***Did you want to die (even a little) when you_____?***<br>***Were you trying to end your life when you _____?***<br>***Or did you think it was possible you could have died from_____?***<br>***Or did you do it purely for other reasons / without ANY intention of killing yourself (like to relieve stress, feel better, get sympathy, or get something else to happen)?*** *(*Self-Injurious Behavior without suicidal intent)<br>If yes, describe: | **Yes** **No**<br>☐ ☐<br><br>Total # of Attempts<br>_____ |
| **Has subject engaged in Non-Suicidal Self-Injurious Behavior?** | **Yes** **No**<br>☐ ☐ |

| | |
|---|---|
| **Interrupted Attempt:**<br>When the person is interrupted (by an outside circumstance) from starting the potentially self-injurious act *(if not for that, actual attempt would have occurred)*.<br>Overdose: Person has pills in hand but is stopped from ingesting. Once they ingest any pills, this becomes an attempt rather than an interrupted attempt. Shooting: Person has gun pointed toward self, gun is taken away by someone else, or is somehow prevented from pulling trigger. Once they pull the trigger, even if the gun fails to fire, it is an attempt. Jumping: Person is poised to jump, is grabbed and taken down from ledge. Hanging: Person has noose around neck but has not yet started to hang - is stopped from doing so.<br>***Has there been a time when you started to do something to end your life but someone or something stopped you before you actually did anything?***<br>If yes, describe: | **Yes** **No**<br>☐ ☐<br>Total # of interrupted<br>______ |
| **Aborted Attempt:**<br>When person begins to take steps toward making a suicide attempt, but stops themselves before they actually have engaged in any self-destructive behavior. Examples are similar to interrupted attempts, except that the individual stops him/herself, instead of being stopped by something else.<br>***Has there been a time when you started to do something to try to end your life but you stopped yourself before you actually did anything?***<br>If yes, describe: | **Yes** **No**<br>☐ ☐<br>Total # of aborted<br>______ |
| **Preparatory Acts or Behavior:**<br>Acts or preparation towards imminently making a suicide attempt. This can include anything beyond a verbalization or thought, such as assembling a specific method (e.g., buying pills, purchasing a gun) or preparing for one's death by suicide (e.g., giving things away, writing a suicide note).<br>***Have you taken any steps towards making a suicide attempt or preparing to kill yourself (such as collecting pills, getting a gun, giving valuables away or writing a suicide note)?***<br>If yes, describe: | **Yes** **No**<br>☐ ☐ |
| **Suicidal Behavior:**<br>Suicidal behavior was present during the assessment period? | **Yes** **No**<br>☐ ☐ |
| **Suicide:** | **Yes** **No**<br>☐ ☐ |
| ***Answer for Actual Attempts Only*** | Most Lethal Attempt Date: |
| **Actual Lethality/Medical Damage:**<br>0. No physical damage or very minor physical damage (e.g., surface scratches).<br>1. Minor physical damage (e.g., lethargic speech; first-degree burns; mild bleeding; sprains).<br>2. Moderate physical damage; medical attention needed (e.g., conscious but sleepy, somewhat responsive; second-degree burns; bleeding of major vessel).<br>3. Moderately severe physical damage; *medical* hospitalization and likely intensive care required (e.g., comatose with reflexes intact; third-degree burns less than 20% of body; extensive blood loss but can recover; major fractures).<br>4. Severe physical damage; *medical* hospitalization with intensive care required (e.g., comatose without reflexes; third-degree burns over 20% of body; extensive blood loss with unstable vital signs; major damage to a vital area).<br>5. Death | *Enter Code*<br>______ |
| **Potential Lethality: Only Answer if Actual Lethality=0**<br>Likely lethality of actual attempt if no medical damage (the following examples, while having no actual medical damage, had potential for very serious lethality: put gun in mouth and pulled the trigger but gun fails to fire so no medical damage; laying on train tracks with oncoming train but pulled away before run over).<br>0 = Behavior not likely to result in injury<br>1 = Behavior likely to result in injury but not likely to cause death<br>2 = Behavior likely to result in death despite available medical care | *Enter Code*<br>______ |
